# Kinesin-14 family proteins and microtubule dynamics define *S. pombe* mitotic and meiotic spindle assembly, and elongation

**DOI:** 10.1242/jcs.240234

**Published:** 2020-06-08

**Authors:** Ana Loncar, Sergio A. Rincon, Manuel Lera Ramirez, Anne Paoletti, Phong T. Tran

**Affiliations:** 1 Institute Curie, PSL Research University, CNRS, UMR 144, F-75005 Paris, France; 2 Instituto de Biología Funcional y Genómica/Departamento de Microbiología y Genética, Consejo Superior de Investigaciones Científicas (CSIC)/Universidad de Salamanca, Salamanca 37007, Spain; 3 University of Pennsylvania, Department of Cell and Developmental Biology, Philadelphia, PA 19104, USA

**Keywords:** Spindle, Mitosis, Meiosis, Microtubule, Kinesin, Fission yeast

## Abstract

To segregate the chromosomes faithfully during cell division, cells assemble a spindle that captures the kinetochores and pulls them towards opposite poles. Proper spindle function requires correct interplay between microtubule motors and non-motor proteins. Defects in spindle assembly or changes in spindle dynamics are associated with diseases, such as cancer or developmental disorders. Here, we compared mitotic and meiotic spindles in fission yeast. We show that, even though mitotic and meiotic spindles underwent the typical three phases of spindle elongation, they have distinct features. We found that the relative concentration of the kinesin-14 family protein Pkl1 is decreased in meiosis I compared to mitosis, while the concentration of the kinesin-5 family protein Cut7 remains constant. We identified the second kinesin-14 family protein Klp2 and microtubule dynamics as factors necessary for proper meiotic spindle assembly. This work defines the differences between mitotic and meiotic spindles in fission yeast *Schizosaccharomyces pombe*, and provides prospect for future comparative studies.

This article has an associated First Person interview with the first author of the paper.

## Abstract (precis)

**Highlighted Article:** Chromosome segregation requires the spindle apparatus in both mitosis and meiosis. We report that kinesin-14 family proteins and microtubule dynamics function differently in mitosis and meiosis.

## INTRODUCTION

Cell division is an essential feature of all living organisms. Mitosis allows cell renewal and amplification by producing two daughter cells of the same ploidy as the mother cell. Meiosis promotes genetic mixing within species by producing four gametes of halved ploidy (Page and Hawley, 2003). In both cases, faithful chromosome segregation is crucial for a successful outcome of cell division. To accomplish this, cells build a spindle – a complex molecular machine that comprises microtubules (MTs), motor proteins, non-motor MT-associated proteins (MAPs) and other regulatory proteins.

As it is well recognized that irregularities in spindle dynamics can result in chromosome segregation errors, potentially leading to cancer, congenital diseases and cell death (Holland and Cleveland, 2009; Thompson et al., 2013), knowing the similarities and/or differences in spindle dynamics between mitosis and meiosis in the same organism may help our understanding of these processes (Ohkura, 2015). Fission yeast *Schizosaccharomyces pombe* is a simple, genetically tractable organism with many of its genes conserved in humans. Importantly, fission yeast can complete mitosis and meiosis in the same environment. This allows robust simultaneous study of both processes and provides an opportunity to understand the intrinsic differences in their spindle dynamics.

**Fig. 1.**
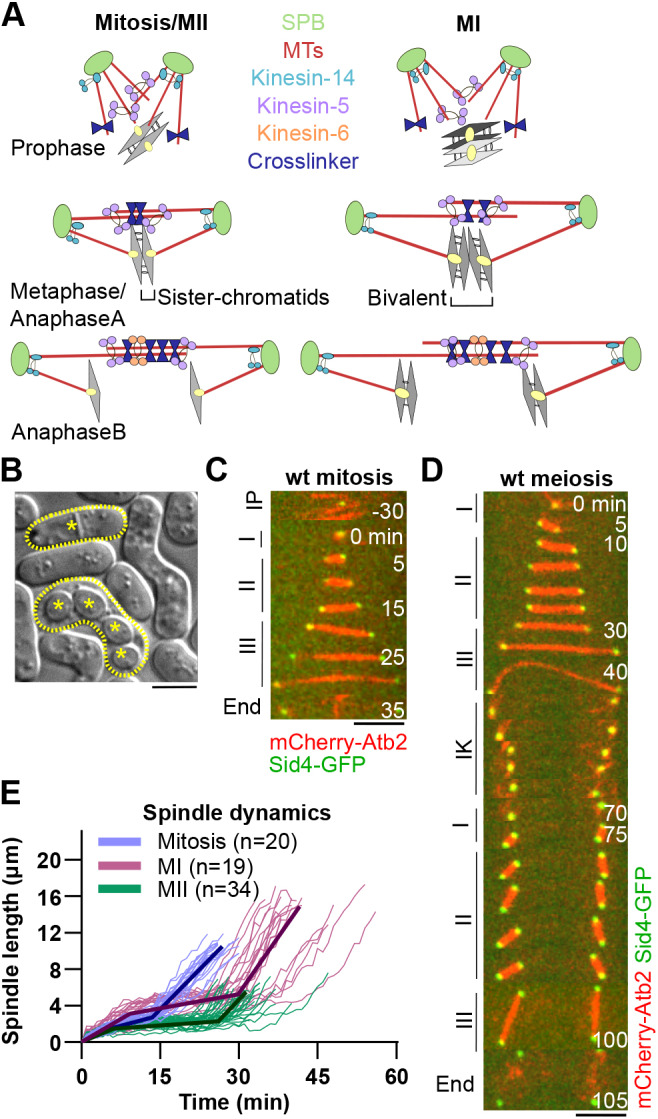
**Fission yeast mitotic and meiotic spindles** **have distinct dynamics.** (A) Model of spindle assembly and elongation in mitosis, MI and MII. (B) DIC image of fission yeast cells. A *wt* haploid cell post mitosis (surrounded by a dashed line, asterisk indicating the septum) is shown above a *wt* diploid zygote post MI and MII (surrounded by dashed line, asterisks indicating the four round spores) in the same field of view. Scale bar: 5 μm. (C) Time-lapse image of *wt* cell expressing mCherry-Atb2 (α-tubulin) and Sid4-GFP (SPB component) from interphase (IP; 30 min prior to spindle formation) until spindle breakdown. Phases of mitotic spindle assembly and elongation are indicated on the left (I, phase I; II, phase II; III, phase III). Frames were taken at 5-min intervals. Scale bar: 5 µm. (D) Time-lapse images of *wt* zygotes expressing mCherry-Atb2 and Sid4-GFP from spindle assembly in MI until spindle disassembly during MII. Phases of meiotic spindle assembly and elongation are indicated on the left side. Frames were taken at 5-min intervals. Scale bar: 5 µm. (E) Analysis of spindle dynamics. Plotted is *wt* spindle length during mitosis (*n*=20), MI (*n*=19) and MII (*n*=34) over time. Bold curves represent the averaged spindle for each phase.

In fission yeast, the initial assembly of the spindle occurs in phase I, during prophase/prometaphase, when nucleation of MTs from the spindle pole body (SPB, equivalent of centrosome) starts inside the nucleus (Fig. 1A). MTs are anchored to the SPBs via their minus-ends, which are bundled and focused by the kinesin-14 family protein Pkl1 (Grishchuk et al., 2007; Syrovatkina and Tran, 2015; Yukawa et al., 2015). Plus-ends of MTs originating from the opposite SPB grow and interact through motors and MAPs. Spindle bipolarity is achieved when interdigitating MTs are crosslinked and organized into antiparallel arrays by the kinesin-5 family protein Cut7 (Hagan and Yanagida, 1992; Kapitein et al., 2005; Sharp et al., 1999), inducing the separation of spindle poles. Spindle bipolarity then ensures the segregation of genetic material to the opposite sides of the cell. The bipolar spindle grows in length until metaphase/anaphase A (phase II), when a constant spindle length is reached and maintained by a ‘force-balance’ mechanism (Goshima et al., 2005; Saunders and Hoyt, 1992; Syrovatkina et al., 2013). The forces involved in maintaining the spindle length during chromosome congression originate from the spindle midzone (kinesin-5 family proteins), the spindle poles (kinesin-14 family proteins, dynein), the kinetochores (kinesin-8 family proteins) and the cohesion between sister chromatids (cohesin) (Pavin and Tolić, 2016; Saunders and Hoyt, 1992). When all kinetochores are captured by MTs and the chromosomes are bi-oriented (sister-kinetochores are attached to the opposite poles), the spindle assembly checkpoint (SAC) is satisfied (Musacchio and Salmon, 2007). This activates an endopeptidase separase which abolishes the cohesion between the sister-chromatids and triggers chromatid disjunction (Uhlmann et al., 1999; Yanagida, 2000). In anaphase B (phase III), the spindle elongates, thus segregating the chromatids into two daughter cells that keep the same ploidy as the mother cell.

Haploid fission yeast cells can undergo mitosis or, in nutrient-poor conditions, cells of opposite mating type can fuse and form a zygote (Fig. 1B). A zygote undergoes two meiotic divisions and produces four quiescent haploid spores. Meiotic spindle assembly is preceded by the so-called nuclear horsetail movement – long-range oscillations led by the SPB, which promote meiotic recombination (Chikashige et al., 1994; Miki et al., 2002; Yamamoto et al., 1999). Different than in mitosis, homologous chromosomes are linked through reciprocal recombination (chiasmata) and form bivalents during the first meiotic division (MI). Owing to specific meiotic cohesion and an altered kinetochore architecture (Hauf et al., 2007; Sakuno et al., 2009; Watanabe and Nurse, 1999; Yokobayashi and Watanabe, 2005), the homologous chromosomes in the bivalent ‘mono-orient’, i.e. sister-kinetochores attach to the same pole. This, together with step-wise removal of cohesion by separase (Kitajima et al., 2004; Miyazaki et al., 2017), results in segregation of homologs into daughter cells and halved ploidy within the two nuclei produced at the end of MI. In the second meiotic division (MII), kinetochores have a mitosis-like architecture, resulting in the segregation of sister-chromatids into daughter cells, similar to what is seen in mitosis. The four haploid nuclei produced by the end of MII are finally encapsulated into spores by growth of the forespore membrane around them (Nakamura-Kubo et al., 2003).

In most organisms, including fission yeast, deficiency of kinesin-5 family proteins leads to monopolar spindles during mitosis and failure to segregate the chromosomes (Goshima et al., 2005; Hagan and Yanagida, 1990; Kapoor et al., 2000; Olmsted et al., 2014). It has recently been reported that force-balance and spindle bipolarity are restored in fission yeast cells that are simultaneously defective of kinesin-5 family protein Cut7 and kinesin-14 family protein Pkl1 (Olmsted et al., 2014; Rincon et al., 2017; Yukawa et al., 2018). Here, we show that, in contrast to what happens during mitosis, fission yeast zygotes deficient in Cut7 and Pkl1 failed to assemble a functional MI spindle, and instead persisted to stay in a monopolar state. Whereas the total amount of all spindle components tested by us increased from mitosis to MI, the total amount of Pkl1 remained constant. Additionally, the second fission yeast kinesin-14 family protein Klp2 antagonized Cut7 during MI and its deletion rescued the Cut7-independent bipolar spindle assembly. Finally, we report that MTs were more dynamic in zygotes than in vegetative cells. This suggests altered regulation of MT dynamics between mitosis and meiosis, which also impacts the ability to assemble a functional spindle in the absence of Cut7 and Pkl1. Our data point out precise adaptations in meiotic spindle dynamics that fine-tune chromosome segregation, despite similarities with mitotic spindles.

## RESULTS

### Spindle dynamics differ during mitosis and meiosis in fission yeast

To assess spindle dynamics in mitosis and meiosis, we imaged wild-type (*wt*) fission yeast cells expressing the α-tubulin Atb2 tagged with mCherry (mCherry-Atb2) and the SPB component Sid4 tagged with green fluorescent protein (Sid4-GFP), cultured in malt-extract (ME) medium to induce mating. Fission yeast MTs reorganized at the end of vegetative interphase (IP) to build a mitotic spindle, with typical three-phase dynamics (Fig. 1C) (Nabeshima et al., 1998). Mitotic spindle studies were traditionally performed in yeast extract medium supplemented with Leu, Ura, Ade, His and Lys (YE5S). Therefore, we checked if mitotic spindle dynamics are comparable in YE5S and ME medium (Fig. S1). We found that they are similar, except that the spindle elongation velocity in phase III is slightly slower in cells undergoing mitosis in ME medium, which results in ∼10% longer duration of mitosis (YE5S: 27±2 min; ME: 30±3 min). In zygotes (Fig. 1D), MI and MII spindles exhibited a three-phase progression similar to mitosis. MI spindle disassembly was followed by interkinesis, a phase of rest between two meiotic divisions where MTs are cytoplasmic and undergo dynamic instability, analogous to vegetative interphase. Two MII spindles then formed synchronously in phase I of MII, and meiosis ended with four distinctly separated SPBs. Parameters defining spindle dynamics during mitosis, MI and MII are provided in Table 1.

**Table 1.**
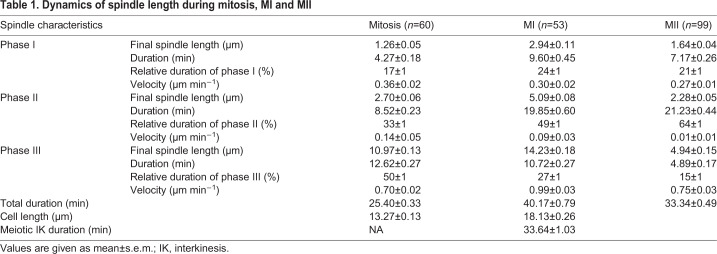
**Dynamics of spindle length during mitosis, MI and MII**

The final spindle lengths at each of the three phases, as well as the duration of phases, differed between mitosis, MI and MII. We next measured spindle dynamics over time (Fig. 1E). MI final spindle length was increased by 25% on average compared to the length of mitotic spindles, and MI spindle elongation velocity in phase III was faster than in mitosis. In contrast to vegetative mitotic cells, zygotes spent the largest proportion of spindle elongation time in phase II of MI and MII. Finally, zygotes spent a similar proportion of time in phase I of MI and MII, but the velocity of spindle assembly in zygotes was lower than in mitotic cells. These differences in spindle dynamics implied qualitative and/or quantitative changes in spindle components, such as motors and MAPs, or changes in MT dynamics between mitosis and meiosis.

### Chromosome segregation is severely compromised in *cut7Δpkl1Δ* zygotes

**Fig. 2.**
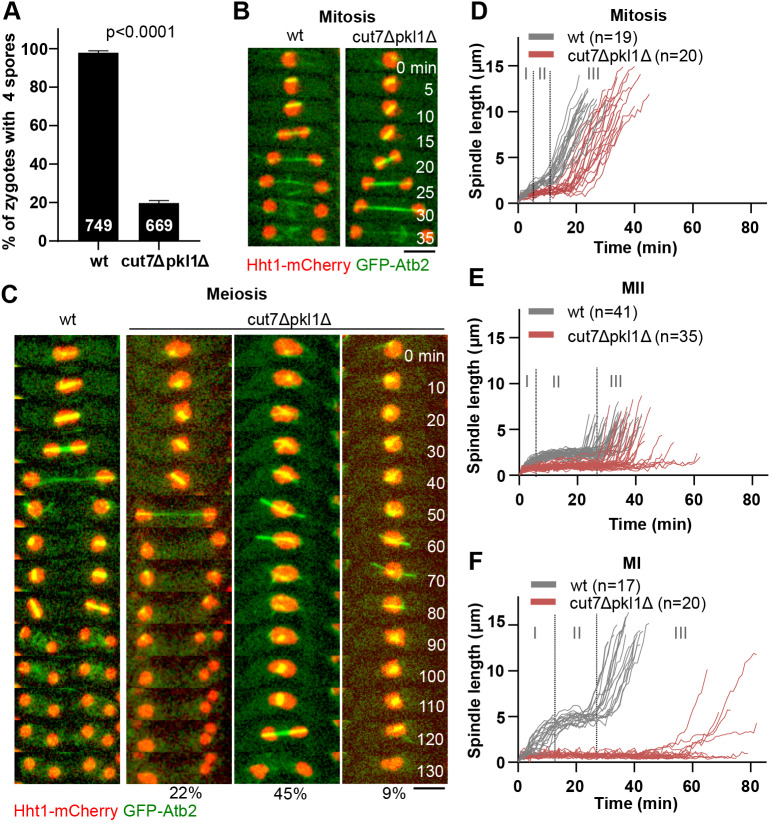
**Spindles in *cut7Δpkl1Δ* cells fail to segregate chromosomes during meiosis.** (A) Bar graph comparing the number of *wt* (*n*=749) and *cut7Δpkl1Δ* (*n*=669) zygotes producing the typical four spores. The *P*-value was calculated by χ² test. (B) Time-lapse images of *wt* and *cut7Δpkl1Δ* mitotic cells expressing GFP-Atb2 (α-tubulin) and Hht1-mCherry (histone H3) from spindle assembly until spindle breakdown. Frames were taken at 5-min intervals. Scale bar: 5 µm. (C) Time-lapse images of *wt* and *cut7Δpkl1Δ* zygotes expressing GFP-Atb2 and Hht1-mCherry from spindle assembly in MI until spindle breakdown in MII. Percentages below the three *cut7Δpkl1Δ* phenotypes represent their frequency (*n*=54). Frames were taken at 10-min intervals. Scale bar: 5 µm. (D) Analysis of mitotic spindle dynamics. Plotted are *wt* (*n*=19) versus *cut7Δpkl1Δ* (*n*=20) spindle lengths during mitosis. Dotted vertical gray lines indicate a shift between phases (I, II, III) for *wt* spindles. (E) Analysis of spindle dynamics during MII. Plotted are *wt* (*n*=41) versus *cut7Δpkl1Δ* (*n*=35) spindle lengths. Dotted vertical gray lines indicate a shift between phases (I, II, III) for *wt* spindles. (F) Analysis of spindle dynamics during MI. Plotted are *wt* (*n*=17) and *cut7Δpkl1Δ* (*n*=20) MI spindle lengths. Dotted vertical gray lines indicate a shift between phases (I, II, III) for *wt* spindles.

Kinesin-14 Pkl1 and kinesin-5 Cut7 are antagonistic motors involved in mitotic spindle assembly. Whereas Cut7 deletion leads to monopolar spindles and is non-viable (Hagan and Yanagida, 1990), the simultaneous deletion of Cut7 and Pkl1 results in the formation of a short bipolar spindle in mitosis, capable of segregating the chromosomes (Rincon et al., 2017; Syrovatkina and Tran, 2015; Yukawa et al., 2018). However, when these mutants were mated, their zygotes failed to produce the typical four spores (20±1% zygotes with four spores; Fig. 2A, Fig. S2A), consistent with a previous report (Shirasugi and Sato, 2019). This was suggestive of a problem in meiotic chromosome segregation. We imaged *wt* and *cut7Δpkl1Δ* cells expressing GFP-Atb2 and Hht1-mCherry (histone H3) to visualize chromosome segregation. Although the mitotic spindle in *cut7Δpkl1Δ* cells was capable of segregating the DNA into two separate pools of equal size (Fig. 2B), meiotic chromosome segregation was perturbed and three distinct mutant phenotypes in *cut7Δpkl1Δ* meiosis were detected (Fig. 2C). The first subset of zygotes managed to segregate the DNA mass in MI but one of the MII spindles failed to complete DNA separation, resulting in three DNA masses upon MII completion (22% of zygotes). In the second subset of zygotes, the spindle appeared to elongate, as if the zygotes entered phase III of MI, but without segregating the DNA mass. A spindle assembled again, this time separating the DNA into two separate masses (45% of zygotes). Finally, in the third subset, the spindle completely failed to separate the DNA mass, resulting in one DNA mass at the end of meiosis (9% of zygotes). These observed errors in chromosome segregation might explain the abnormal number of spores at the end of meiotic divisions.

### Spindle integrity is compromised specifically during MI of *cut7Δpkl1Δ* zygotes

To study spindle dynamics during meiosis of *cut7Δpkl1Δ* cells, we imaged *cut7Δpkl1Δ* zygotes expressing mCherry-Atb2 and Sid4-GFP. Contrary to mitosis, in which all of the *cut7Δpkl1Δ* cells assembled a bipolar spindle, *cut7Δpkl1Δ* zygotes failed to do so in meiosis (Fig. S2B-D): 74±2% of *cut7Δpkl1Δ* zygotes failed to establish a bipolar spindle and separate the SPBs during MI, consistent with a previous report (Shirasugi and Sato, 2019). In addition, among the zygotes that had assembled bipolar spindles in MI, 8±2% failed to assemble a spindle in MII. Unsuccessful SPB separation in MII might result from errors during the preceding MI, or from the intrinsic properties of *cut7Δpkl1Δ* MII spindles.

In *wt* zygotes, there are two SPBs at the end of MI – which duplicate, such that there are four SPBs in MII (Fig. 1D). In the *cut7Δpkl1Δ* zygotes that have initially failed to assemble a spindle, we observed a second spindle assembly onset accompanied by an increase in the number of SPBs (Fig. S2C, far right panel). An increase in SPB number after MI spindle failure could indicate that SPBs in *cut7Δpkl1Δ* zygotes were duplicated twice, and that *cut7Δpkl1Δ* zygotes entered MII despite failure of chromosome segregation in MI. We confirmed this result by imaging *wt* and *cut7Δpkl1Δ* zygotes expressing Cdc13 (also known as cyclin-B) tagged to GFP (Cdc13-GFP) and mCherry-Atb2. In *wt* zygotes, Cdc13-GFP localization was visible in the nucleus, on the spindle and SPBs (Fu et al., 2009; Izawa et al., 2005). Upon onset of phase III in MI, the Cdc13-GFP signal was partially degraded (Fig. S2E). The pool of Cdc13-GFP was replenished upon MII phase I onset, and was degraded again upon phase III onset of MII. Similar dynamics of Cdc13-GFP were visible in *cut7Δpkl1Δ* zygotes that formed monopolar spindles, except that Cdc13-GFP degradation relative to the onset of spindle assembly was delayed by 16 min on average in *cut7Δpkl1Δ* MI (Fig. S2F). This suggests that the spindle assembly checkpoint (SAC) is active but leaky in *cut7Δpkl1Δ* zygotes, ultimately allowing progression through the meiotic stages regardless of spindle or chromosome segregation defects.

We then compared *wt* and *cut7Δpkl1Δ* spindle dynamics. In mitosis, pre-phase III spindles were shorter and phase III onset was delayed in the *cut7Δpkl1Δ* mutant compared to that in *wt* (Fig. 2D), which is in agreement with previous studies (Rincon et al., 2017; Yukawa et al., 2018). Similar results were observed for MII spindle dynamics (Fig. 2E). In contrast, most MI spindles remained monopolar. The 25% of MI *cut7Δpkl1Δ* spindles that managed to achieve bipolarity and separate the SPBs, did not show the usual three-phase progression of spindle elongation (Fig. 2F), as phase I and II of spindle elongation could not be distinguished. Combined, our data indicate that spindle assembly and elongation are specifically perturbed during MI of *cut7Δpkl1Δ* zygotes.

We next analyzed the polarity of MT protrusions that formed in MI monopolar spindles, by checking the localization of the MT-plus end tracking protein Mal3 (also known as EB1) tagged to GFP (Mal3-GFP) (Busch and Brunner, 2004). The analysis revealed that *cut7Δpkl1Δ* zygotes formed monopolar protrusions with distal MT-plus ends (Fig. S2G). This phenotype is similar to the phenotype of mitotic cells that lack Cut7 (Hagan and Yanagida, 1990).

We conclude that deletion of Pkl1 cannot fully compensate for the absence of Cut7 in MI. This suggests that Cut7 and Pkl1 are differently regulated between mitosis and MI.

### The ratio of Cut7 to Pkl1 is higher in MI than mitosis spindles

To assess whether Cut7 and Pkl1 are, indeed, differently regulated in mitosis and MI, we imaged Cut7 or Pkl1 tagged to GFP together with MTs (mCherry-Atb2) in *wt* mitosis and MI. We first checked the impact of the GFP tag on motor functionality. Cut7 deficiency is lethal in fission yeast (Hagan and Yanagida, 1992), resulting in cell death when Cut7 is inactive or inhibited. Cells expressing Cut7-GFP were viable. Of 45 observed zygotes with Cut7-GFP, 11% showed a collapsed MI spindle, and only one vegetative cell of more than 100 observed showed a monopolar spindle in mitosis. Tagging Cut7 to GFP resulted in less zygotes producing the typical four spores (Fig. S3A; 91% compared to 97% for the strain without the GFP tag), but no difference in growth was observed in serial dilution plate assay (Fig. S3B). Mitotic and MI spindle dynamics of the strain with the Cut7-GFP were comparable to the strain not expressing Cut7-GFP (Fig. S3C,D). These results indicate that the GFP-tagged version of Cut7 was mostly functional.

Pkl1 deficiency has been reported to lead to protrusions in phases II and III in 85% of the mitotic cells grown in YE5S medium (Syrovatkina and Tran, 2015). We observed no protrusions in mitotic cells expressing 3×GFP-tagged Pkl1 grown in YE5S. In ME medium, we observed 13% of mitotic cells with protrusions in phase III and all zygotes showed protrusions in phase III of MI. Despite protrusions in phase III of MI, 3×GFP-tagged Pkl1 had no significant effect on spore production (Fig. S3A; 94% compared to 97% for the strain without GFP-tagged Pkl1) or any consequence on the growth in the serial dilution plate assay (Fig. S3B). Mitotic spindle dynamics of the strain with Pkl1-3×GFP were similar to those of the strain without GFP-tagged Pkl1 (Fig. S3E), whereas MI spindle dynamics showed a longer phase II duration and a shorter phase II spindle in MI (Fig. S3F). The results indicate that Pkl1-3×GFP is mostly functional.

Despite slight changes regarding spindle dynamics, as well as the observed protrusions and decreased percentage of zygotes with four spores that suggested a partial but not complete loss of functionality, we observed no changes in phase I. Therefore, we concluded that Cut7-GFP and Pkl1-3×GFP are suitable to investigate localization and recruitment of Cut7 and Pkl1 during phase I in both mitosis and MI.

**Fig. 3.**
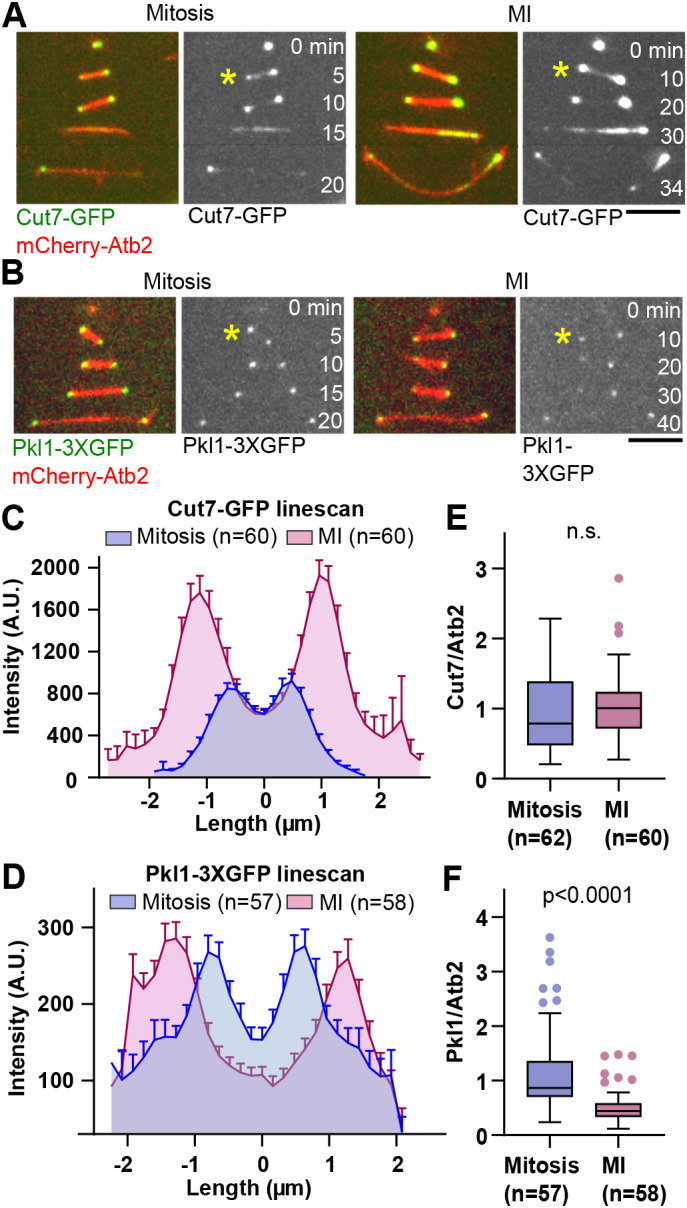
**The relative concentration of the kinesin-14 family protein Pkl1 is lower in MI than in mitosis.** (A) Time-lapse images of cells during mitosis and MI expressing mCherry-Atb2 (α-tubulin) and the kinesin-5 family protein Cut7 tagged to GFP (Cut7-GFP). Asterisks indicate the spindle at transition between phase I and phase II, 1 min before the spindle reaches a constant length in phase II. Scale bar: 5 µm. (B) Time-lapse images of cells during mitosis and MI expressing mCherry-Atb2 and kinesin-14 Pkl1 tagged to 3×GFP (Pkl1-3×GFP). Asterisks indicate the spindle at transition between phase I and II, 1 min before the spindle reaches a constant length in phase II. Scale bar: 5 µm. (C) Analysis of Cut7-GFP signal distribution on the spindle at transition between phase I and II in mitosis (*n*=60) and MI (*n*=60). A.U., arbitrary unit. (D) Analysis of Pkl1-3×GFP signal distribution on the spindle at transition between phase I and II in mitosis (*n*=57) and MI (*n*=58). (E) Box-and-whisker plot comparing the relative concentration of Cut7-GFP per spindle MTs (Cut7/Atb2) at transition between phase I and phase II during mitosis (*n*=62) and MI (*n*=60). n.s., not significant. (F) Box-and-whisker plot comparing the relative concentration of Pkl1-3×GFP per spindle MTs (Pkl1/Atb2) at transition between phase I and phase II during mitosis (*n*=57) and MI (*n*=58). The *P*-value was calculated by using Mann–Whitney test.

Similar to previous observations by Hagan and Yanagida (1992), Cut7-GFP localized to the spindle and spindle poles from spindle assembly to spindle disassembly during both mitosis and MI (Fig. 3A). Similarly, the localization pattern of Pkl1-3×GFP appeared unchanged between MI and mitosis. Pkl1-3×GFP localized to the spindle poles shortly after spindle assembly had started, and remained associated with SPBs until spindle disassembly. Pkl1-3×GFP was also detected on the spindle during phase I to phase II transition (Fig. 3B).

We then measured Cut7-GFP and Pkl1-3×GFP signal distribution along the spindles during phase I to phase II transition by line scan analysis, 1 min before the spindle reached a constant length that marks the beginning of phase II.

Both Pkl1-3×GFP and Cut7-GFP displayed maximum intensity peaks at the start and end of the line scan, which corresponds to the spindle poles. Cut7-GFP maximum intensity was considerably higher in MI (Fig. 3C), whereas Pkl1-3×GFP maximum intensity was similar in mitosis and MI (Fig. 3D). Moreover, total intensity of Cut7-GFP, calculated by integrating the intensity profiles from the line scan, was elevated in MI compared to that in mitosis, whereas total intensity of Pkl1-3×GFP remained similar in both types of spindle (Fig. S3G,H). Because the total MT intensity is different in mitotic and MI spindles (Fig. S3I), we normalized the intensity values obtained for Cut7-GFP and Pkl1-3×GFP to the MT intensity values. This way we found that the relative concentration of Cut7 remained unchanged (Fig. 3E), whereas the relative concentration of Pkl1-3×GFP per MT was lower in MI than in mitotic spindles (Fig. 3F). This might explain why *pkl1* deletion in MI, unlike in mitosis, cannot compensate for deletion of *cut7*, and suggests that an additional factor counteracts Cut7 activity during phase II of MI to maintain force-balance.

### Kinesin-14 Klp2 function is distinctive in MI and mitosis spindles

**Fig. 4.**
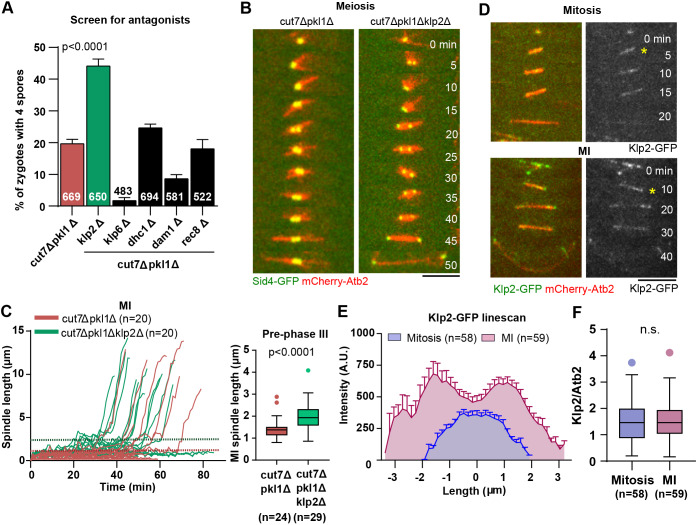
**Deletion of the kinesin-14 family protein Klp2 partially rescues bipolar spindle defects of *cut7Δpkl1Δ* zygotes during MI.** (A) Bar graph comparing the number of spores of *cut7Δpkl1Δ* (*n*=669), and the triple mutants *cut7Δpkl1Δklp2Δ* (*n*=650), *cut7Δpkl1Δklp6Δ* (*n*=483), *cut7Δpkl1Δdhc1Δ* (*n*=694), *cut7Δpkl1Δdam1Δ* (*n*=581) and *cut7Δpkl1Δrec8Δ* (*n*=522) zygotes. Zygotes *cut7Δpkl1Δ* and *cut7Δpkl1Δklp2Δ* are represented by red and green bars, respectively; triple mutants that did not show a rescue in the number of zygotes producing four spores are represented by black bars. The indicated *P*-value was calculated by χ² test. (B) Time-lapse images of *cut7Δpkl1Δ* and *cut7Δpkl1Δklp2Δ* zygotes expressing mCherry-Atb2 (α-tubulin) and Sid4-GFP (SPB component) from spindle assembly until spindle breakdown in MI. Frames were taken at 5-min intervals. Scale bar: 5 µm. (C) Analysis of spindle dynamics during MI. Plotted are *cut7Δpkl1Δ* (*n*=20) versus *cut7Δpkl1Δklp2Δ* (*n*=20) MI spindle lengths. Horizontal dotted lines indicate the maximum lengths of pre-phase III spindles for *cut7Δpkl1Δ* (red) and *cut7Δpkl1Δklp2Δ* (green). The box-and-whisker plot on the right compares the final length of pre-phase III spindles in *cut7Δpkl1Δ* (*n*=24) and *cut7Δpkl1Δklp2Δ* (*n*=29). The *P*-value was calculated by using Mann–Whitney test. (D) Time-lapse images of cells expressing mCherry-Atb2 and Klp2-GFP. Asterisks indicate the spindle at transition between phase I and II, 1 min before the spindle reaches a constant length in phase II. Scale bar: 5 µm. (E) Analysis of Klp2-GFP signal distribution on the spindle at transition between phase I and II in mitosis (*n*=58) and MI (*n*=59). (F) Box-and-whisker plot comparing the relative concentration of Klp2-GFP per spindle MTs at transition between phase I and phase II during mitosis (*n*=58) and MI (*n*=59). n.s., not significant.

To identify additional spindle components that could counteract Cut7-dependent forces in meiosis (Hagan and Yanagida, 1990; Syrovatkina et al., 2013), we screened other motors and MAPs reported to produce forces in the spindle (Syrovatkina et al., 2013). We reasoned that, if a protein antagonizes Cut7, its deletion in the *cut7Δpkl1Δ* background should restore bipolar spindle assembly in MI. This, in turn, would result in proper chromosome segregation and increase the percentage of zygotes producing the typical four spores. We systematically deleted candidate motors and MAPs in the *cut7Δpkl1Δ* background, mated the triple mutants and scored for spore number (Fig. 4A). Of all the proteins tested (kinesin-14 family protein Klp2, kinesin-8 family protein Klp6, dynein Dhc1, kinetochore protein Dam1, and meiotic cohesin Rec8), only the deletion of kinesin-14 Klp2 resulted in zygotes that produced the typical four spores more frequently, i.e. 44±2% of *cut7Δpkl1Δklp2Δ* zygotes produced four spores compared to 20±1% in the *cut7Δpkl1Δ* background. Next, we turned to live cell imaging of *cut7Δpkl1Δklp2* zygotes to determine how *klp2* deletion confers an increase in the number of zygotes producing four spores.

Live cell imaging of *cut7Δpkl1Δklp2Δ* zygotes revealed that they succeeded to build a bipolar spindle in MI capable of separating the SPBs (Fig. 4B). Whereas only 26±2% of *cut7Δpkl1Δ* zygotes managed to establish spindle bipolarity and separate SPBs, 49±2% of *cut7Δpkl1Δklp2Δ* zygotes assembled bipolar spindles in MI (Fig. S4A). We next compared mitotic and MI spindle dynamics in the triple mutant. MI spindle dynamics comparison revealed that, not only is spindle bipolarity re-established but the usual three-phase spindle elongation dynamics are partially rescued (Fig. 4C). This differed substantially from *cut7Δpkl1Δ* zygotes, in which the three-phase spindle dynamics was not observed (Figs 2F and 4C).

It has recently been reported that deletion of *klp2* partially rescues *cut7Δpkl1Δ* mitotic spindle dynamics, indicating that Klp2 produces inward forces alongside Pkl1, thereby counteracting the Cut7-dependent forces (Yukawa et al., 2018). Our mitotic spindle analyses showed that *klp2* deletion partially rescues the time needed to reach phase III – but it did not rescue phase III spindle length in *cut7Δpkl1Δ* cells (Fig. S4B-E). This result was unlike the one noticed in *cut7Δpkl1Δklp2Δ* MI spindles, whose pre-phase III spindle length was partially restored (Fig. 4C). Together, these results suggest that kinesin-14 Klp2 counteracts Cut7-dependent outward pushing forces and represents a new player in the force-balance maintenance in phase II of MI spindles.

Klp2 is a minus-end directed motor that can crosslink and slide MTs, thereby affecting spindle length (Carazo-Salas et al., 2005; Janson et al., 2007; Syrovatkina et al., 2013; Troxell et al., 2001). We tagged Klp2 with GFP to study its localization in mitotic and MI spindles. Tagging Klp2 had no effect on serial dilution assay results when compared with those using the non-tagged strain (Fig. S4F) and showed a non-significant decrease in the percentage of zygotes producing four spores (Fig. S4G; 94% compared to 97% without GFP tag). The velocity of spindle elongation in phase III of mitotic spindle dynamics was faster and phase II duration was increased in the strain with tagged Klp2 (Fig. S4H). Tagging Klp2 resulted in a longer phase II spindle in MI (Fig. S4I) and two of the 17 observed zygotes required more time to reach spindle bipolarity. These results indicate that Klp2-GFP is mostly functional.

Imaging of Klp2-GFP showed that it localized along the spindle, starting from spindle assembly until early phase III in both mitosis and MI (Mana-Capelli et al., 2012; Yukawa et al., 2018) (Fig. 4D). Line scan analysis of Klp2-GFP showed higher maximum intensity peaks in MI, indicating an increase in the total amount of Klp2-GFP in MI compared to that in mitosis (Fig. 4E). However, the normalized concentration of Klp2-GFP relative to spindle MTs remained unchanged (Fig. 4F). We also noticed that Klp2-GFP localized more intensely at the spindle poles in MI compared to its localization in mitosis. This is probably not an artifact of mitosis and MI spindle length difference, as the mitotic Pkl13×GFP signal was clearly observed as two distinct peaks in line scan analysis (Fig. 3D), regardless of the shorter mitotic spindle.

### Suppressing MT dynamics restored MI spindle bipolarity in *cut7Δpkl1Δ* zygotes

Considering that *klp2* deletion rescued spindle bipolarity only partially in the *cut7Δpkl1Δ* zygotes, we searched for additional factors that participate in force-balance in MI phase II spindles. We considered that MT dynamics might be differently regulated between mitosis and meiosis. Current imaging limitations prevented us from measuring individual MT dynamics within the spindle, where there are many crosslinked MTs; instead, we measured individual MT bundle dynamics before onset of mitosis (interphase), and in the pause period between meiotic MI and MII (interkinesis). We compared the dynamics of individual MT bundles that grew straight and originated from SPBs. Parameters defining MT dynamics during interphase and interkinesis are provided in Table 2.

**Table 2.**
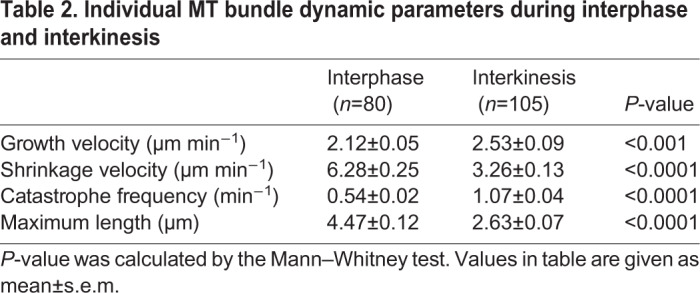
**Individual MT bundle dynamic parameters during interphase and interkinesis**

**Fig. 5.**
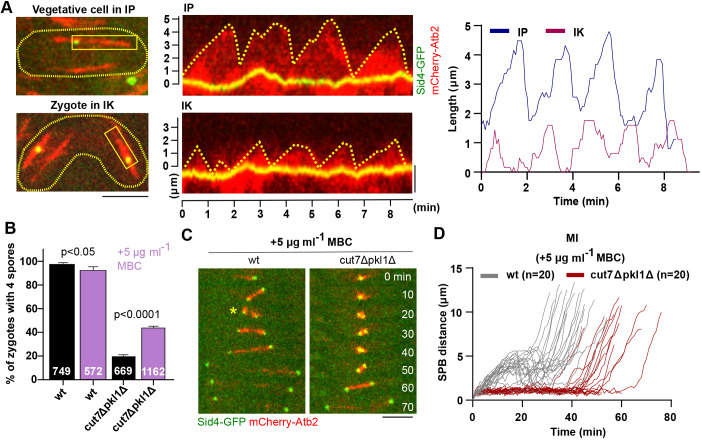
**Inhibition of MT polymerization restores MI spindle bipolarity in *cut7Δpkl1Δ* zygotes.** (A) Images show a vegetative cell (top) and a representative zygote (bottom), each surrounded by a dashed line, expressing mCherry-Atb2 (α-tubulin) and Sid4-GFP (SPB component). Scale bar: 5 µm. Kymographic images were taken of the boxed regions containing the SPB and MT bundle in the zygote during interkinesis (IK) and in the vegetative cell during interphase (IP). The kymographic images in the middle show representative IK- and IP-MT bundle dynamics over time. Scale bar: 2 µm. Analysis comparing the differences between IP- and IK-MT bundle dynamics are shown on the right. (B) Quantification of the number of zygotes producing the typical four spores in the presence of 5 µg ml^−1^ MBC (*wt n*=572, *cut7Δpkl1Δ n*=1162) and without MBC (*wt n*=749, *cut7Δpkl1Δ n*=669). The *P*-value was calculated by using χ² test. (C) Time-lapse images of *wt* and *cut7Δpkl1Δ* zygotes expressing mCherry-Atb2 and Sid4-GFP from spindle assembly until spindle breakdown during MI. The asterisk indicates a collapsed metaphase spindle. Frames were taken at 10-min intervals. Scale bar: 5 µm. (D) Analysis of spindle dynamics. Plotted are SPB distances of *wt* (*n*=20) and *cut7Δpkl1Δ* (*n*=20) cells during MI in the presence of 5 µg ml^−1^ MBC.

Kymographs and plots of MT bundle dynamics in a vegetative cell, and a zygote are shown in Fig. 5A. MT bundles began their growth at the SPB and grew continuously until they abruptly entered catastrophe, by switching to rapid shortening, and shrank back to the SPB. We found that MT bundles shrink slower and grow faster in interkinesis but reach a decreased maximum length compared with bundles in interphase. Additionally, interkinesis MT bundles underwent catastrophe twice as often as interphase MT bundles. In general, meiotic interkinesis MTs are more dynamic than mitotic interphase MTs. Although not measured within the spindles, these results on interphase and interkinesis MT bundles suggest that MT dynamics are differently regulated between mitotic cells and meiotic zygotes. We, therefore, tested if a modification in MT dynamics influences MI spindle assembly and spore formation in *cut7Δpkl1Δ* zygotes.

We perturbed MT dynamics by using low doses of methyl benzimidazole carbamate (MBC), a drug that inhibits MT polymerization and suppresses MT dynamics (Vela-Corcía et al., 2018; Yenjerla et al., 2009). Mating *wt* zygotes on ME plates with 5 µg ml^−1^ MBC resulted in a slightly decreased frequency of zygotes with four spores (Fig. 5B). In contrast, *cut7Δpkl1Δ* zygotes produced four spores more frequently with the addition of MBC (44±1% in the presence of MBC compared to 20±1% without MBC). Live-cell imaging showed that addition of 5 µg ml^−1^ MBC allowed bipolar spindle assembly in MI within 80±4% of *cut7Δpkl1Δ* zygotes compared to 26±2% of *cut7Δpkl1Δ* zygotes when no MBC was added (Fig. 5C and Fig. S5A).

We also noticed that, in *wt* zygotes treated with MBC, the MI phase II spindle frequently exhibited transient breakage (Fig. 5C, asterisk), a phenotype that was never observed in the *cut7Δpkl1Δ* zygotes. Accordingly, we saw an abrupt decrease of SPB distance in phase II of MI upon spindle breakage (Fig. 5D). However, spindle integrity was subsequently re-established and MI phase III could proceed. Nevertheless, this spindle instability due to the MBC treatment could account for the slightly lower number of *wt* zygotes producing four spores.

*Cut7Δpkl1Δ* spindle dynamics with the addition of MBC showed a delayed onset of MI phase III as well as a shorter MI phase I/phase II spindle, similar to what was observed for *cut7Δpkl1Δ* mitotic progression in the absence of MBC (Fig. 2D). Unlike *cut7Δpkl1Δklp2Δ* (Fig. 4C), *cut7Δpkl1Δ* MI spindles in the presence of 5 µg ml^−1^ MBC did not exhibit a three-phase progression of spindle elongation (Fig. 5D). This suggests that the mechanism of MBC-dependent rescue is distinct from the one that relies on *klp2* deletion.

Finally, addition of low doses of MBC did not seem to impact mitotic progression (Fig. S5B). The relationship between *wt* and *cut7Δpkl1Δ* spindle dynamics with the addition of 5 µg ml^−1^ MBC appeared similar to their relationship without MBC addition (Fig. 2D). We conclude that, in phase II of MI, MT dynamics counteract Cut7-dependent outward pushing forces together with Klp2 to achieve proper force balance and allow the formation of functional bipolar spindles.

## DISCUSSION

In this work, we have analyzed the differences between mitotic and meiotic spindle dynamics in fission yeast. We found that the total amount of MTs, as well as the total amount of molecular motors studied (except the kinesin-14 family protein Pkl1), were higher in MI than in mitosis. In fission yeast, spindle length scales with cell size (Cortés et al., 2018; Krüger et al., 2019). A simple scaling mechanism could, therefore, explain the increase in the final MI spindle lengths during all phases, as well as the increased velocity of MI phase III (Table 1; Krüger et al., 2019). Because zygotes are diploid and larger than haploid vegetative cells (Table 1), they presumably have more MTs, motor proteins and MAPs to organize a spindle. The average 25% increase in MI final spindle length compared to mitotic spindles could be readily explained by the increased cell volume and increased availability of spindle components.

We further found that in MI, unlike mitosis, the largest proportion of time was spent in phase II (Table 1). In MI, the homologous chromosomes in the bivalent are held together by cohesin and chiasmata (Kitajima, 2003; Molnar et al., 2003; Watanabe and Nurse, 1999). The temporal requirement to complete the resolution of chiasmata could explain why phase II of MI is prolonged compared to mitosis.

Finally, zygotes spent a similar time in phase I of the two meiotic divisions (Table 1), but the velocity of spindle elongation in phase I of MI was decreased compared to that in mitosis, indicating that it might not increase with cell size, unlike in phase III. This decreased phase I velocity might result from a modification in MT dynamics (Table 2), a hypothesis that needs further testing, as we could not to date access MT dynamics within the spindle for technical reasons.

It has been reported that failure to form a meiotic bouquet leads to aberrant spindles (Tomita and Cooper, 2007). However, imaging of the telomere component Taz1 (tagged to GFP) and the SPB component Sid4 (tagged to mCherry) did not reveal any defect in bouquet formation (Fig. S5C). We have also considered that the kinetochores might not be attached to the spindle MTs in *cut7Δpkl1Δ* MI. Because of the bouquet rearrangement, the kinetochores are far from the SPBs during MI, making it relatively easy to detect kinetochore attachment to MTs. Imaging the kinetochore component Mis12 tagged to GFP and the SPB component Sid4 tagged to mCherry did not reveal a defect in kinetochore attachment (Fig. S5D).

Further, MI spindles have a specific organization of chromosomes in bivalents, with cohesin between the homologous chromosomes, the existence of chiasmata and changes in kinetochore architecture (Molnar et al., 2003; Yokobayashi and Watanabe, 2005; Watanabe and Nurse, 1999; Kitajima et al., 2004). We examined if this organization can influence spindle assembly in the *cut7Δpkl1Δ* background. Individual deletion of the meiosis-specific cohesin Rec8, the meiotic kinetochore protein Moa1, the meiotic sister-chromatid cohesin Rec11 or the meiotic recombination endonuclease Rec12 did neither rescue the *cut7Δpkl1Δ* MI spindle phenotype nor the number of zygotes producing the typical four spores (Fig. S5E). Interestingly, a recent study did report that the double-deletion of Rec12 and Moa1 partially rescued spindle bipolarity in a *cut7Δpkl1Δ* background (Shirasugi and Sato, 2019). These results indicate that chromosome organization can subtly influence mitotic and MI spindle dynamics.

Unlike the other spindle components tested in our work here, the concentration of kinesin-14 Pkl1 relative to spindle MTs is lower in MI than in mitosis (Fig. 3). The regulation behind this decrease of the relative concentration remains unknown. As we have measured the concentration of motor proteins in mitosis and MI by analyzing the fluorescence intensity of GFP attached to them, it is possible that it does not reflect the wild-type state, especially when considering that the GPF-tagged versions Cut7-GFP, Pkl1-3×GFP and Klp2-GFP were not fully functional (Figs S3,S4). Nevertheless, by measuring the fluorescence intensities in the same cell but during different stages of cell division, we argue that the differences we observed were, indeed, intrinsic to the type of cell division, and not artifacts due to the fluorescent tag. It has recently been published that, in mouse oocytes, weak overexpression of the kinesin-14 family protein HSET (officially known as KIFC1) during MI accelerates spindle bipolarization during phase I, and results in more focused spindle poles, essentially switching from MI to a more mitotic-like spindle (Bennabi et al., 2018). This, in turn, resulted in aberrant chromosome alignment. It is tempting to think that a reduction of kinesin-14 concentration from mitosis to MI could be evolutionarily conserved.

We identified Klp2 as an additional Cut7 antagonist during MI. It has been demonstrated previously that, in mitosis, Klp2 tethering to SPBs can, to some degree, replace the role of Pkl1 in spindle anchorage and force generation, indicating that in such a situation, Klp2 can effectively oppose Cut7 outward forces (Yukawa et al., 2018). Since Klp2-GFP localized more to the spindle poles during MI than mitosis (Fig. 4E), we propose that Klp2 function is different in MI and mitosis, and that Klp2 is important in the establishment of spindle bipolarity by counteracting the Cut7 outward pushing forces in order to compensate the increase in the ratio between Cut7 and Pkl1. How Klp2 regulation is tuned and which factors influence its functions remain open questions.

Another significant finding of our study is the possibility to restore MI spindle bipolarity in *cut7Δpkl1Δ* by altering MT dynamics with low doses of MBC (Yenjerla et al., 2009; Cortés et al., 2018; Carazo-Salas et al., 2005). Although we could measure parameters of dynamic MT bundles in interkinesis compared to interphase, we were unable to access individual MT dynamics within the spindle. Nevertheless, our results imply changes of spindle MT dynamics during mitosis and meiosis. We hypothesize that MT dynamics can be enhanced in MI phase I and/or phase II compared to mitotic phase I and/or phase II. In any case, the fact that suppression of MT dynamics in *cut7Δpkl1Δ* zygotes restores MI spindle bipolarity and also alters the outcome of *wt* zygote meiosis, but without perturbing mitosis outcome, indicates that MI spindle formation is extremely sensitive to alterations in MT dynamics, and that fine tuning of MT dynamics is crucial for the success of meiosis. However, which specific factors trigger the alteration of MT dynamics during mitosis and meiosis remains to be studied.

In vegetative cells, centromeres are grouped at the nuclear envelope, below the SPB (Funabiki et al., 1993). In zygotes, a telomere bouquet is formed prior to meiotic division, which places telomeres below the SPB and centromeres farther away (Chikashige et al., 1994; Fennell et al., 2015). One possibility is that – because centromeres and kinetochores are less accessible for attachment to MTs, being farther away from the SPBs during MI – an increase in MT dynamics raises the chance of MTs to find and attach to kinetochores (Heald and Khodjakov, 2015; Kirschner and Mitchison, 1986). Moreover, it has been shown that MT pivoting movement around the spindle poles speeds up kinetochore capture during mitosis (Kalinina et al., 2013). Taking into account the decreased concentration of Pkl1 at the spindle poles in MI, it is plausible to assume that MTs are more loosely organized at the SPB and that more MTs are being nucleated during MI (Olmsted et al., 2014). This could result in a greater range of MT pivoting motions, which would increase their chance to encounter and attach to a kinetochore. Indeed, compared to mitosis, where such structures were rarely detected, we observed an abundance of transient and dynamic MTs in the initial stages of spindle assembly during MI (Fig. S5F). These transient dynamic MTs could be newly polymerizing MTs or existing pivoting MTs. The fact that suppression of MT dynamics by addition of MBC rescued spindle bipolarity in *cut7Δpkl1Δ* zygotes, suggests that MT dynamics also contribute to the force-balance establishment within meiotic spindles, possibly by limiting the outward pushing forces generated by MT growth.

Our results open new questions on how motors, MAPs and MT dynamics are modified during mitosis and meiosis. Given the high degree of evolutionarily conserved molecular players involved in spindle assembly, it will be most interesting to expand this study further towards other organisms, to develop our global understanding of mitosis and meiosis.

## MATERIALS AND METHODS

### Production of *S. pombe* mutant strains

All strains used in this work are isogenic to the *S. pombe* wild-type 972 strain*,* and were obtained using genetic crosses, selected by random spore germination and replicated on plates in the presence with appropriate drugs or supplements. All strains are listed in Table S1. Gene deletion and tagging was performed within the endogenous locus, except for Sid4-GFP and Mis12-GFP, which were integrated into the *leu1-32* locus as described previously (Bähler et al., 1998; Keeney and Boeke, 1994). Transformations were performed by using the lithium-DTT method. 25 ml of exponentially growing cells (OD_600nm_=0.5–0.8) were harvested by centrifugation and washed with 10 mM Tris HCl pH 7.4. Cells were centrifuged again and re-suspended in 100 mM lithium acetate supplemented with 10 mM DTT. The suspension was then incubated on an orbital wheel at room temperature for 40 min. Thereafter, 100 µl of suspension was mixed with 80 µl of 100 mM lithium acetate, 10 µl of single-stranded DNA from salmon testes (D9156-5ML, Sigma) and 1 µg of the purified DNA. After 10 min of incubation on an orbital wheel, 300 µl of PEG 4000, dissolved in 100 mM lithium acetate was added. After incubation of 10 min on the wheel, 15 µl of DMSO was added. The mixture was then heat shocked at 42°C for 20 min. Cells were washed with distilled water and plated onto plates containing yeast extract medium supplemented with Leu, Ura, Ade, His, and Lys (YE5S). After 2 d of incubation at 25°C, the plates were replicated on selection plates containing the corresponding selective medium.

### Fission yeast culture

All *S. pombe* strains were maintained at 25°C and cultured in standard media. Generally, two days before imaging, cells were refreshed on a plate containing YE5S. The following day, cells of opposite mating types were mixed on an ME plate, or an ME plate supplemented with 5 µg ml^−1^ MBC, to induce conjugation. The plate with mating cells was incubated at 25°C for 16–20 h before imaging. For spore quantification, the mixture was incubated on an ME plate, or an ME plate supplemented with 5 µg ml^−1^ MBC, at 25°C for two days and subsequently imaged.

Serial dilutions were performed by growing tested strains on a plate containing YE5S at 25°C for 24 h, and then resuspending some of the patch in water so the OD_600nm_ was 1.5. This suspension was serially fourfold diluted, 5 µl of the prepared dilutions was inoculated on plates containing YE5S and then incubated at 25°C or 30°C for 48 or 96 h.

### Live-cell microscopy

For live-cell imaging, cells were put on ME agarose pads, containing 4% agarose (Tran et al., 2004). In experiments where MT dynamics were perturbed, MBC was added to the patch at a final concentration of 5 µg ml^−1^ MBC. Imaging was performed at 25°C. Images were acquired on an inverted Spinning Disk Confocal (Roper/Nikon), equipped with Plan Apochromat 100×/1.4 NA objective lens (Nikon), a PIFOC (perfect image focus) objective stepper, and a charge-coupled device camera (EMCCD 512×512 QuantEM; Photometrics).

For spindle dynamics, stacks of seven planes spaced by 1 µm were acquired for each channel with 200 ms exposure for 561 nm wavelength and 100 ms exposure for 491 nm wavelength, binning one, and an electronic gain of 3 for both wavelengths. For each time-lapse movie, each stack was taken every minute for a duration of 180–240 min.

For intensity measurements, stacks of 11 planes spaced by 0.5 µm were acquired for each channel with 100 ms exposure, binning one, and electronic gain of 1 or 3. For each time-lapse movie, each stack was taken every minute for a duration of 90 min.

For MT bundle dynamics, stacks of seven planes spaced by 1 µm were acquired for each channel with 100 ms exposure for 561 nm wavelength and 100 ms exposure for 491 nm wavelength, binning one, and an electronic gain of 3 for both wavelengths. For each time-lapse movie, each stack was taken every 5 s for a duration of 30 min.

To quantify spores, bright-field images of zygotes were taken at random, and the spores inside the zygotes counted.

### Image analysis

Analyses were performed by Metamorph 7.8 and ImageJ 1.52i. Maximum projections of each stack were performed for analysis of spindle dynamics and for presentation unless stated otherwise; sum projections were performed for intensity measurements.

Spindle dynamics were determined by measuring the duration of the mCherry-Atb2 fluorescence signal between Sid4-GFP signals over time or, when 5 µg ml^−1^ MBC was added, by measuring the distance between SPBs, from the onset of spindle assembly to spindle breakdown by using a semi-automated MATLAB program.

Signal intensity measurements were performed by line scan analysis, reading out the average intensity per pixel, subtracting the background and adding all positive values to obtain the total amount of fluorescence intensity.

MT bundle dynamics were approximated by tracing the shape of mCherry-Atb2 signal on a kymograph, and calculating the slopes of the gathered curves to obtain the velocity of MT bundle growth and shrinkage. The origins and the endings of the curves were approximated by extending the curves to the edges of the SPB signal. The movements of the SPB were accounted and corrected for in our measurements. The catastrophe frequency was approximated as a reciprocal value of time the MT bundle spent in elongation.

#### Feature detection

Microscopy images were analyzed with a semi-automated MATLAB program that can find features in microscopy images. After the computer finds the features, they are presented to the user, and the user can manually correct them. In this work, the program was used to detect SPBs and spindles in maximal projections of confocal microscopy stacks. Features are represented as the coordinates of two points (SPBs) or a polygonal chain (spindle). The scripts for the analysis are integrated in a Graphical User Interface, and the complete version of the program is available upon request. We provide the MATLAB scripts for feature extraction, and describe the pipeline of the analysis:

#### Cell segmentation

Initially, the user segments a cell manually in the relevant frames of the movie. Only the selected frames, and the area inside the segmented region of interest is considered for feature detection for a cell. Features are detected for each of the selected frames.

#### Spindle detection – 1

Short spindles (<4 μm) are found by applying a 2 pixel Gaussian blur to the image, and subsequently thresholding applying Otsu's method as provided in MATLAB's *multithresh*. Serial thresholding steps are applied until the area above the threshold contains <10% of the pixels of the region defined by the user as the cell. After that, regions above the threshold are merged using a convex hull algorithm as provided in MATLAB's *bwconvhull* and the coordinates of the pixels above the threshold are fit to a first degree polynomial by regression. The resulting line is uniformly sampled such that points are distributed along its length at 1 pixel distance, and constitutes the spindle feature. Once a spindle is found to be >4 μm, spindles are detected using the method explained below.

#### Spindle detection – 2

This method relies on the fact that long spindles are often aligned with the major axis of the cell. Therefore, the distance, with respect to the major axis of the cell, is very similar in neighboring pixels that belong to the spindle. A maximal projection of the image is made along the major axis of the region defined by the user as the cell; for each projected pixel, the position along the major axis [X], the intensity [I], and distance with respect to the major axis [Y] are stored in 1-dimensional vectors. To keep only the pixels belonging to the spindle, subsequent discarding steps are applied: a moving standard deviation filter of window size 3 pixels is applied to [Y], and positions with a standard deviation value >3 pixels, are discarded. Next, ‘groups’ – denoting a series of consecutive non-discarded pixels – containing <4 pixels are discarded. Pixels for which the intensity in [I] is <20% of the maximum pixel in the cell are discarded. For the remaining groups, if the distance along the major axis [X] between the closest points of two consecutive groups divided by the difference in distance from the major axis [Y] between those two points is >1.5, the group that is furthest from the center of the cell is discarded. The curve defined by [Y] is smoothened by applying a moving median filter of window size 10, resulting in [Y_2_]. Then, [X,Y_2_] coordinates are fitted to a first-order polynomial when the cumulative distance along the curve [X,Y_2_] is <8 μm, and to a third-order polynomial otherwise. The resulting curve is sampled as described previously, and constitutes the spindle feature.

#### Detection of SPBs

The ten most-intense pixels inside a cell are considered for the analysis. If two or more distinct regions are present in the image, the coordinates of the centroids of the two biggest regions are returned. If there is only one region (often when Spindle Pole Bodies are too close), the pixel with the weakest signal is removed from the analysis recursively until two distinct regions form, and the coordinates of the centroids of the two regions are returned. If this method fails, the coordinates of the two most intense pixels in the cell are returned.

#### Measurements

Spindle length is defined by using the *N* coordinates of the spindle polygonal chain [*x*_1_, *x*_2_, …, *x_N_*] and [*y*_1_, *y*_2_, …, *y_N_*], as the sum of Eucledian distances between consecutive points:
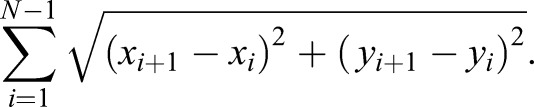
SPB separation is defined as the Euclidean distance between the two points that define the spindle pole bodies.

### Quantification and statistics

All graphs and box plots were generated using GraphPad 8.0 (Prism). All bar graph and line graph data are shown as mean values with error bars representing ±s.e.m. Data in box and whisker graphs were obtained by using the Tukey method; black lines shows the median value. Analysis of statistical significance was performed by using Mann–Whitney or χ² tests. Obtained *P*-values are shown within plots and data sets were defined as significantly different when *P*<0.05. *n* values represent the number of cells and are shown inside the plots. All experiments were performed at least three times.

## Supplementary Material

Supplementary information

Reviewer comments
