# Supplementary material for: Kinesin-14 family proteins and microtubule dynamics define S. pombe mitotic and meiotic spindle assembly, and elongation
Source: J Cell Sci. 2020 Jun 8;133(11):jcs240234. doi: 10.1242/jcs.240234 (PMC7295595; doi:10.1242/jcs.240234)
Supplement: Supplementary information [file joces-133-240234-s1.pdf]

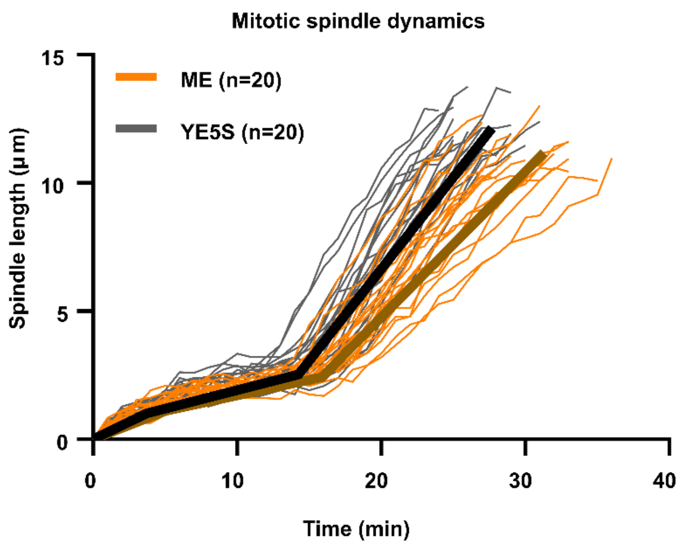

Fig. S1. Mitotic dynamics are comparable in YE5S and ME media. Comparative plot of *wt* spindle dynamics in YE5S (n=20) and ME (n=20). Bold curves represent the average spindle.

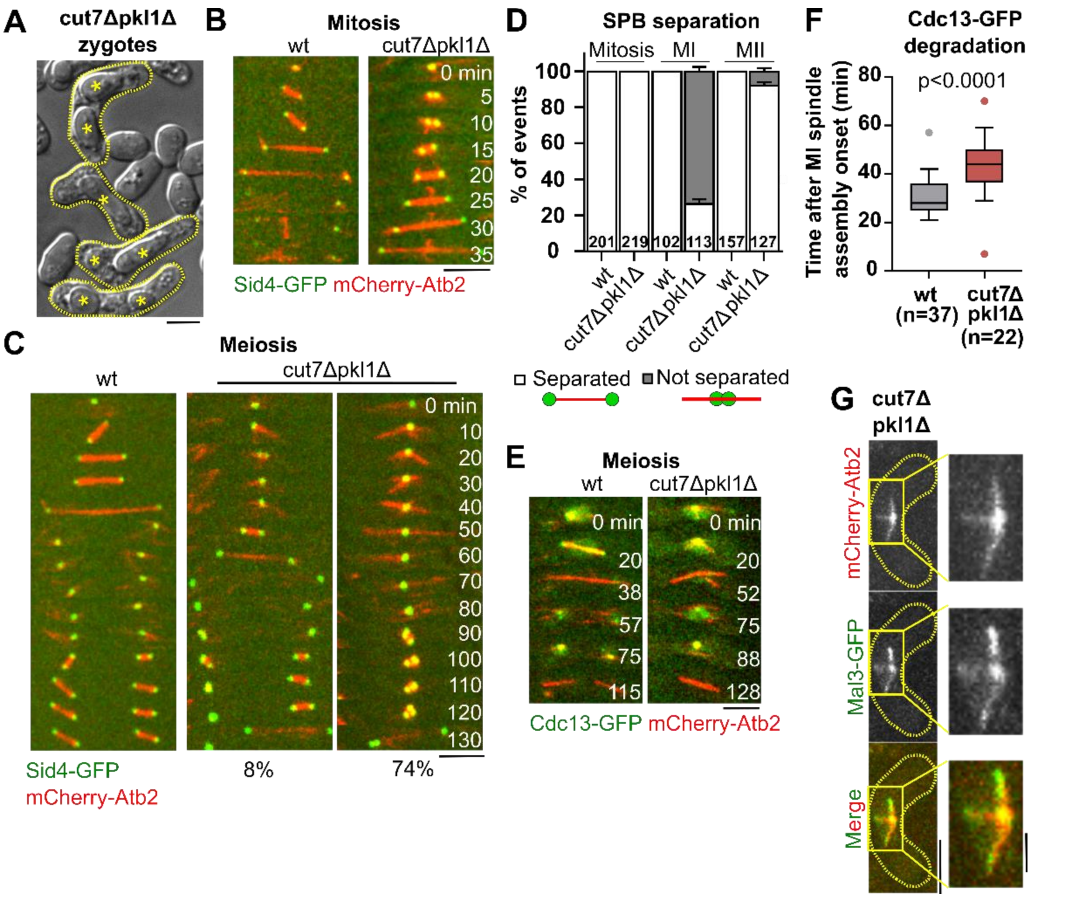

Fig. S2. ***Cut7Δpkl1Δ*** **zygotes fail to establish a bipolar spindle in MI, but still enter MII. (A)** DIC image of *cut7Δpkl1Δ* zygotes. Yellow lines indicate zygotes with abnormal spores (asterisks). **(B)** Time-lapse images of *wt* and *cut7Δpkl1Δ* vegetative cells expressing mCherry-Atb2 and Sid4-GFP from spindle assembly to spindle breakdown. Scale bar, 5 µm. **(C)** Time-lapse images of *wt* and *cut7Δpkl1Δ* zygotes expressing mCherry-Atb2 and Sid4-GFP from spindle assembly in MI to spindle breakdown in MII. Percentages below the two *cut7Δpkl1Δ* phenotypes represent their frequency (n=113). Scale bar, 5 µm. **(D)** Bar graph comparison of SPB separation in mitosis (*wt* n=201, *cut7Δpkl1Δ* n=219), MI (*wt* n=102, *cut7Δpkl1Δ* n=113), and MII (*wt* n=157, *cut7Δpkl1Δ* n=127). **(E)** Time-lapse images of *wt* and *cut7Δpkl1Δ* zygotes expressing mCherry-Atb2 and Cdc13-GFP (cyclin-B). Scale bar, 5 µm. **(F)** Box-and-whisker plot comparison of Cdc13-GFP degradation time after spindle assembly onset in MI of *wt* (n=37) and *cut7Δpkl1Δ* (n=22) zygotes. **(G)** Representative image of a *cut7Δpkl1Δ* zygote in phase I/phase II of MI expressing mCherry-Atb2 and Mal3-GFP. Scale bar, 5 µm. For insets, scale bar is 2 µm.

Journal of Cell Science • Supplementary information

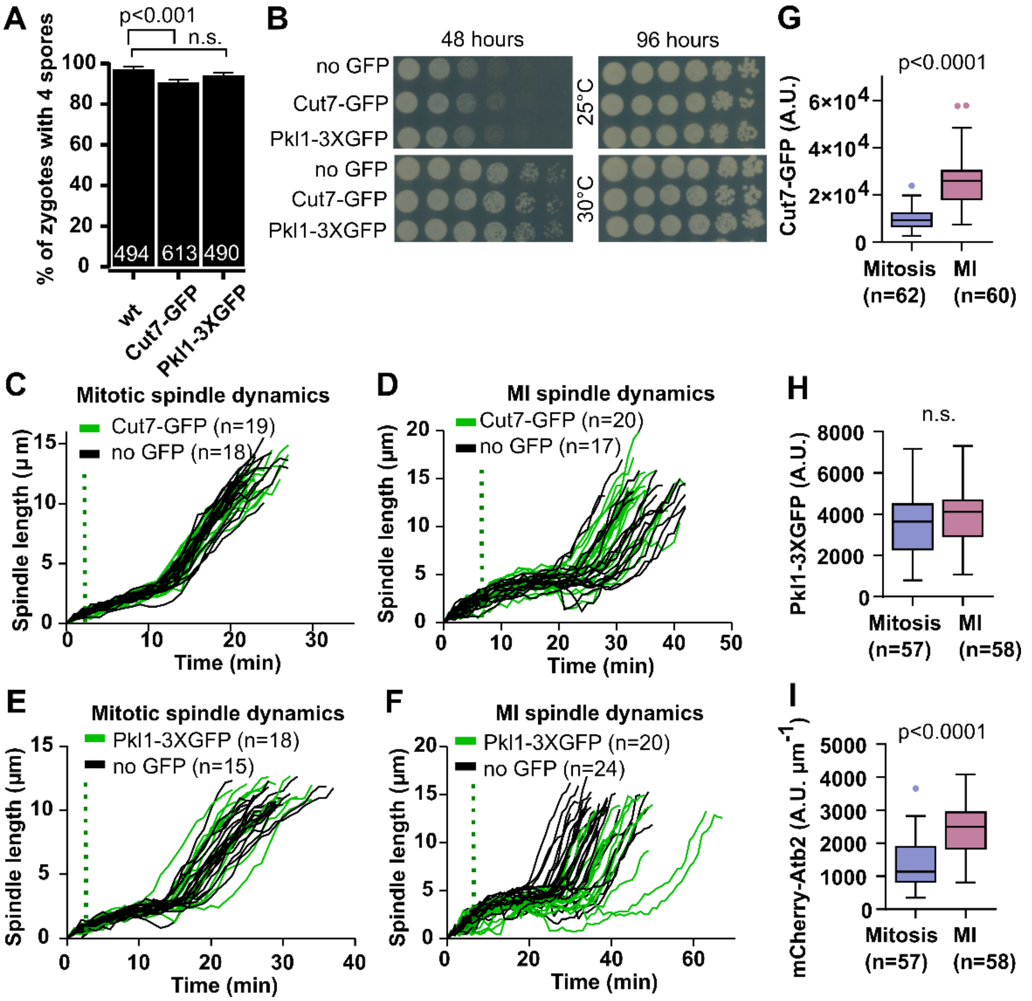

Fig. S3. Total amount of Pkl1 remains constant from mitosis to MI. (A) Bar-graph comparison of the number of zygotes producing the typical four spores in strains without GFP (n=494), in strains with Cut7-GFP (n=613), and in strains with Pkl1-3XGFP (n=490). p-values were calculated by χ^2 test. **(B)** Serial dilution (fourfold) plate assay showing growth of strains without GFP, and strains with Cut7-GFP or Pkl1-3XGFP. Plates were incubated 48 or 96 hours at the indicated temperatures. **(C)** Comparative plot of mitotic spindle length dynamics in the strain without GFP (n=18) and in the strain with Cut7-GFP (n=19). The green vertical dotted line represents a time point in phase I/phase II transition when the intensity of Cut7-GFP was measured. **(D)** Comparative plot of MI spindle length dynamics in the strain without GFP (n=17) and in the strain with Cut7-GFP (n=20). The green vertical dotted line represents a time point in phase I/phase II transition when the intensity of Cut7-GFP was measured. **(E)** Comparative plot of mitotic spindle length dynamics in the strain without GFP (n=15) and in the strain with Pkl1-3XGFP (n=18). The green vertical dotted line represents a time point in phase I/phase II transition when the intensity of Pkl1-3XGFP was measured. **(F)** Comparative plot of MI spindle length dynamics in strains without GFP (n=24) and in strains with Pkl1-3XGFP (n=20). The

Journal of Cell Science • Supplementary information

green vertical dotted line represents a time point in phase I/phase II transition when the intensity of Pkl1-3XGFP was measured. **(G)** Box-and-whisker plot comparison of Cut7-GFP total intensity in mitosis (n=62) and MI (n=60). **(H)** Box-and-whisker plot comparison of Pkl1-3XGFP total intensity in mitosis (n=57) and MI (n=58). **(I)** Box-and-whisker plot comparison of MT total intensity per spindle length in mitosis (n=57) and MI (n=58). p-values were calculated by Mann-Whitney test.

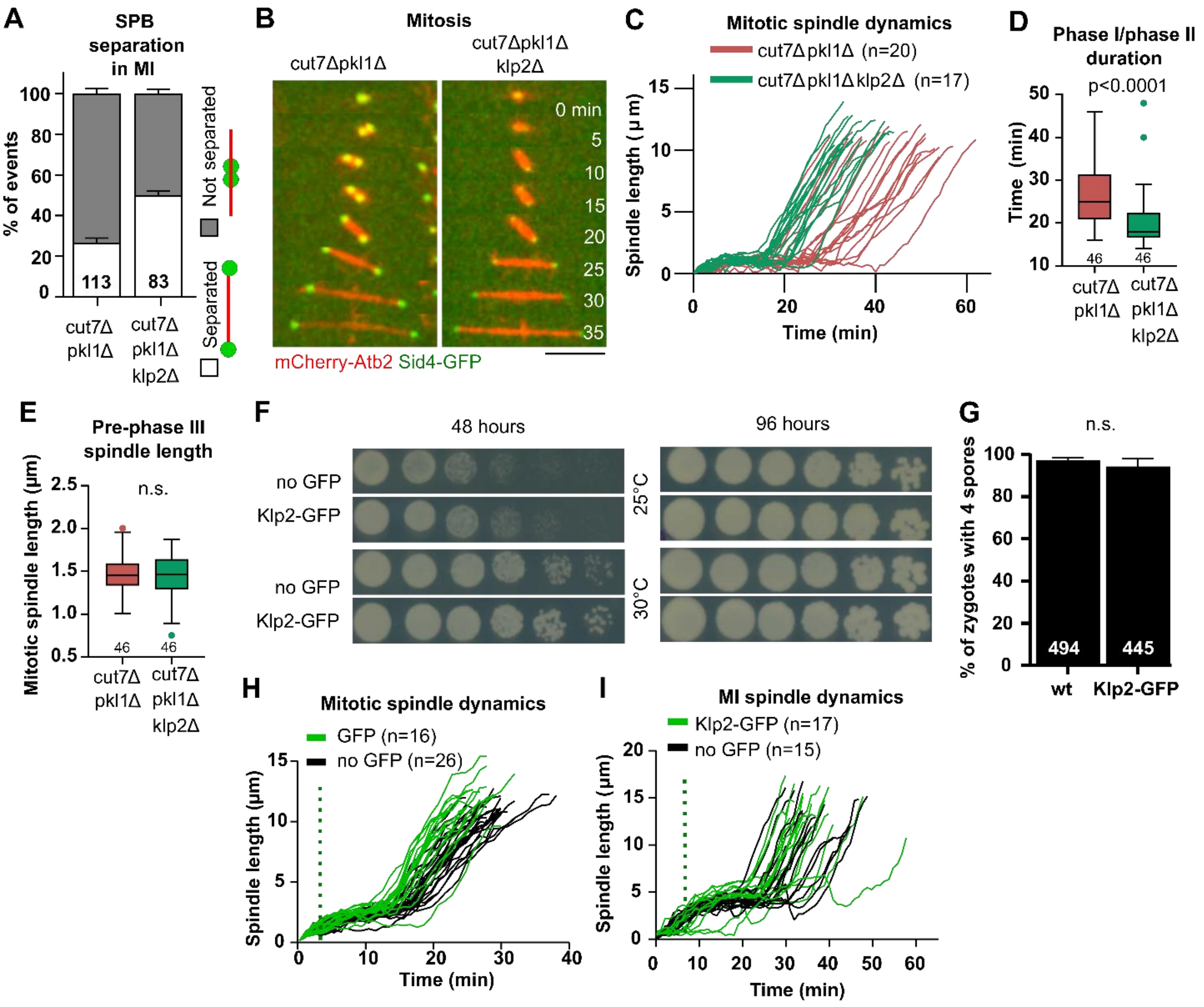

Fig. S4. *Klp2* deletion rescues metaphase duration, but not final metaphase spindle length in *cut7Δpkl1Δ* mitosis. (A) Bar graph comparison of SPB separation in *cut7Δpkl1Δ* (n=113) and *cut7Δpkl1Δklp2Δ* (n=83) MI. **(B)** Time-lapse images of *cut7Δpkl1Δ* and *cut7Δpkl1Δklp2Δ* mitotic cells expressing mCherry-Atb2 and Sid4-GFP from spindle assembly to spindle disassembly. Scale bar, 5 µm. **(C)** Comparative plot of *cut7Δpkl1Δ* (n=20) and *cut7Δpkl1Δklp2Δ* (n=17) mitotic spindle length dynamics. **(D)** Box-and-whisker plot comparison of mitotic phase I/phase II duration in *cut7Δpkl1Δ* (n=46) and *cut7Δpkl1Δklp2Δ* (n=46). **(E)** Box plot comparison of pre-phase III mitotic spindle length of *cut7Δpkl1Δ* (n=46) and *cut7Δpkl1Δklp2Δ* (n=46). p-values were calculated by Mann-Whitney test. **(F)** Serial dilution (fourfold) plate assay showing growth of strain without GFP, and strain with Klp2-GFP. Plates were incubated 48 or 96 hours at the indicated temperatures. **(G)** Bar-graph comparison of the number of zygotes producing the typical four spores in strains without GFP (n=494) and with Klp2-GFP (n=445). p-values were calculated by χ^2 test. **(H)** Comparative plot of mitotic spindle length dynamics in the strain without GFP (n=26) and in the strain with Klp2-GFP (n=16). The green vertical dotted line represents an average time point in phase I/phase II transition when the

Journal of Cell Science • Supplementary information

intensity of Klp2-GFP was measured. **(I)** Comparative plot of MI spindle length dynamics in the strain without GFP (n=15) and in strains with Klp2-GFP (n=17). The green vertical dotted line represents a time point in phase I/phase II transition when the intensity of Klp2-GFP was measured.

Journal of Cell Science • Supplementary information

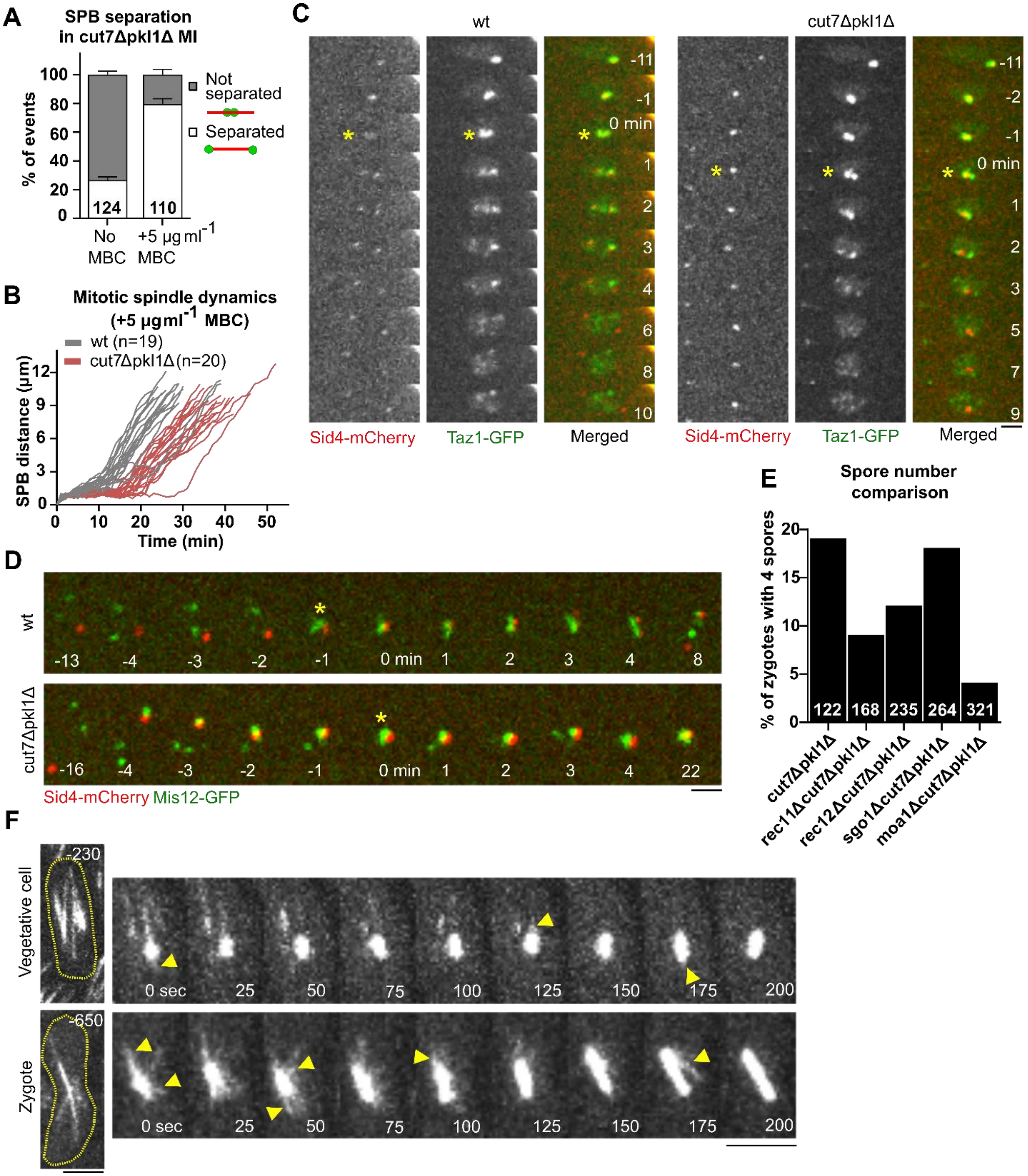

Fig. S5. Chromosome organization and architecture are not the cause of failed *cut7Δpkl1Δ* meiosis. (A) Bar graph comparison of SPB separation in *cut7Δpkl1Δ* zygotes with (n=110) and without addition of 5 µg ml^{-1} MBC (n=124). **(B)** Comparative plot of *wt* (n=19) and *cut7Δpkl1Δ* (n=20) mitotic spindle dynamics with the addition of 5 µg ml^{-1} MBC. **(C)** Kymographs of *wt* and *cut7Δpkl1Δ* zygotes expressing Sid4-mCherry and a telomere component Taz1-GFP. Yellow asterisks indicate a time point in which

Journal of Cell Science • Supplementary information

telomeres start to dissociate from the SPB. Scale bar, 2 µm. **(D)** Kymographs of *wt* and *cut7Δpkl1Δ* zygotes expressing Sid4-mCherry and a kinetochore component Mis12-GFP. Yellow asterisks indicate a time point in which all the kinetochores are pulled to the SPB. Scale bar, 2 µm. **(E)** Bar graph comparison of the number of *cut7Δpkl1Δ* (n=122), *cut7Δpkl1Δrec11Δ* (n=168), *cut7Δpkl1Δrec12Δ* (n=235), *cut7Δpkl1Δsgo1Δ* (n=264), and *cut7Δpkl1Δmoa1Δ* zygotes producing the typical four spores. **(F)** Spindle MTs in mitotic and MI from spindle assembly onset in phase I. Yellow arrows indicate potential pivoting MTs or transient new polymerizing MTs. Time indicated on the images is in seconds. Scale bar, 5 µm. For insets, scale bar is 2 µm.

Table S1: *S. pombe* Strain List

Identifier	Genotype	Source
AP240	*h- ade6-M210 ura4-D18 leu1-32*	Lab collection
AP241	*h+ ade6-M210 ura4-D18 leu1-32*	Lab collection
TP2872	*h- cut7::Nat pkl1::ura4+ ade6-M210 ura4-D18 leu1-32*	Lab collection
TP2873	*h+ cut7::Nat pkl1::ura4+ ade6-M210 ura4-D18 leu1-32*	Lab collection
TP2927	*h- mCherry-Atb2:Hph leu1:Sid4-GFP leu1-32 ura4-D18*	Lab collection
TP2944	*h+ mCherry-Atb2:Hph leu1:Sid4-GFP leu1-32 ura4-D18*	Lab collection
TP3793	*h- GFP-Atb2:Hph Hht1-mCherry:Kan ade6-M210 ura4-D18 leu1-32*	This study
TP3794	*h+ GFP-Atb2:Hph Hht1-mCherry:Kan ade6-M210 ura4-D18 leu1-32*	This study
TP3653	*h- Hht1-mCherry:Kan GFP-Atb2:Hph cut7::Nat pkl1::ura4+ ade6-M210 ura4-D18 leu1-32*	This study
TP3654	*h+ Hht1-mCherry:Kan GFP-Atb2:Hph cut7::Nat pkl1::ura4+ ade6-M210 ura4-D18 leu1-32*	This study
TP2491	*h- cut7::Nat pkl1::ura4+ mCherry-Atb2:Hph Sid4-GFP:Kan leu1-32 ura4-D18*	Lab collection
TP2492	*h+ cut7::Nat pkl1::ura4+ mCherry-Atb2:Hph Sid4-GFP:Kan leu1-32 ura4-D18*	Lab collection
TP3453	*h+ Cdc13-GFP:Kan mCherry-Atb2:Hph ade6-M210 ura4-D18 leu1-32*	Lab collection
TP3454	*h- Cdc13-GFP:Kan mCherry-Atb2:Hph ade6-M210 ura4-D18 leu1-32*	Lab collection
TP4090	*h- Cdc13-GFP:Kan cut7::Nat pkl1::ura4+ mCherry-Atb2:Hph ade6-M210 ura4-D18 leu1-32*	This study
TP4091	*h+ Cdc13-GFP:Kan cut7::Nat pkl1::ura4+ mCherry-Atb2:Hph ade6-M210 ura4-D18 leu1-32*	This study
TP4224	*h- cut7::Nat pkl1::ura4+ Mal3-linker-GFP:Kan mCherry-Atb2::Hph ura4-D18 leu1-32*	This study
TP4225	*h+ cut7::Nat pkl1::ura4+ Mal3-linker-GFP:Kan mCherry-Atb2::Hph ura4-D18 leu1-32*	This study
TP116	*h- mCherry-Atb2::Hph ura4-D18 leu1-32*	Lab collection
TP319	*h+ mCherry-Atb2::Hph ura4-D18 leu1-32*	Lab collection
TP2508	*h- Pkl1-3xGFP:Nat mCherry-Atb2:Hph leu1-32 ura4-D18*	Lab collection
TP3834	*h+ Pkl1-3xGFP:Nat mCherry-Atb2:Hph leu1-32 ura4-D18*	Lab collection
TP2945	*h- Cut7-GFP:Kan mCherry-Atb2:Hph ade6-M210 ura4-D18 leu1-32*	Lab collection
TP2946	*h+ Cut7-GFP:Kan mCherry-Atb2:Hph ade6-M210 ura4-D18 leu1-32*	Lab collection
TP3166	*h- klp2::Kan cut7::Nat pkl1::ura4+ leu1-Sid4-GFP mCherry-Atb2:Hph leu1-32 ura4-D18*	Lab collection
TP3167	*h+ klp2::Kan cut7::Nat pkl1::ura4+ leu1-Sid4-GFP mCherry-Atb2:Hph leu1-32 ura4-D18*	Lab collection
TP3187	*h- dhc1::Kan cut7::Nat pkl1::ura4+ leu1:Sid4-GFP mCherry-Atb2:Hph ade6-M210 ura4-D18 leu1-32*	Lab collection
TP4542	*h+ dhc1::Kan cut7::Nat pkl1::ura4+ leu1:Sid4-GFP mCherry-Atb2:Hph ade6-M210 ura4-D18 leu1-32*	This study
TP3175	*h- dam1::Kan cut7::Nat pkl1::ura4+ mCherry-Atb2:Hph leu1:Sid4-GFP ade6-M210 ura4-D18 leu1-32*	Lab collection
TP4531	*h- dam1::Kan cut7::Nat pkl1::ura4+ mCherry-Atb2:Hph leu1:Sid4-GFP ura4-D18 leu1-32*	This study
TP3710	*h+ cut7::Nat pkl1::ura4+ rec8::Kan mCherry-Atb2:Hph leu1:Sid4-GFP ura4-D18 leu1-32*	This study
TP3891	*h- cut7::Nat pkl1::ura4+ rec8::Kan mCherry-Atb2:Hph leu1:Sid4-GFP ade6-M210 ura4-D18 leu1-32*	Lab collection
TP3905	*h- Klp2-GFP:ura4+ mCherry-Atb2:Hph ura4-D18 leu1-32*	Lab collection
TP4537	*h+ Klp2-GFP:ura4+ mCherry-Atb2:Hph ura4-D18 leu1-32*	This study
TP4445	*h+ Taz1-GFP:Kan Sid4-mCherry:Hph*	This study
TP4446	*h- Taz1-GFP:Kan Sid4-mCherry:Hph*	This study
TP4298	*h+ cut7::Nat pkl1::Kan Taz1-GFP:Kan Sid4-mCherry:Hph*	This study
TP4299	*h+ cut7::Nat pkl1::Kan Taz1-GFP:Kan Sid4-mCherry:Hph*	This study
TP4218	*h+ rec11::Kan cut7::Nat pkl1::ura4+ mCherry-Atb2:Hph leu1:Sid4-GFP*	This study
TP4219	*h- rec11::Kan cut7::Nat pkl1::ura4+ mCherry-Atb2:Hph leu1:Sid4-GFP*	This study

Journal of Cell Science • Supplementary information

TP4221	*h+ rec12::Kan cut7::Nat pkl1::ura4+ mCherry-Atb2:Hph leu1:Sid4-GFP*	This study
TP4222	*h- rec12::Kan cut7::Nat pkl1::ura4+ mCherry-Atb2:Hph leu1:Sid4-GFP*	This study
TP4229	*h- sgo1::Kan cut7::Nat pkl1::ura4+ leu1:Sid4-GFP mCherry-Atb2:Hph*	This study
TP4230	*h+ sgo1::Kan cut7::Nat pkl1::ura4+ leu1:Sid4-GFP mCherry-Atb2:Hph*	This study
TP4539	*h+ moa1::Kan cut7::Nat pkl1::ura4+ mCherry-Atb2:Hph leu1:Sid4-GFP*	This study
TP4538	*h- moa1::Kan cut7::Nat pkl1::ura4+ mCherry-Atb2:Hph leu1:Sid4-GFP*	This study
TP1511	*h+ Mis12-GFP:leu2+ Sid4-mCherry:Hph ura4-D18 leu1-32*	Lab collection
TP2943	*h- Sid4-mCherry:Hph Mis12-GFP:leu2+ ura4-D18 leu1-32*	Lab collection
TP3223	*h- cut7::Nat pkl1::ura4+ Sid4-mCherry:Hph Mis12-GFP:leu2+ ade6- ura4-D18 leu1-32*	Lab collection
TP3062	*h+ cut7::Nat pkl1::ura4+ Sid4-mCherry:Hph Mis12-GFP:leu2+ ade6- ura4-D18 leu1-32*	Lab collection

Journal of Cell Science • Supplementary information
