# Supplementary material for: Kinesin-14 family proteins and microtubule dynamics define S. pombe mitotic and meiotic spindle assembly, and elongation
Source: J Cell Sci. 2020 Jun 8;133(11):jcs240234. doi: 10.1242/jcs.240234 (PMC7295595; doi:10.1242/jcs.240234)
Supplement: Reviewer comments [file joces-133-240234_review_history.pdf]

Kinesin-14 family proteins and microtubule dynamics define *S. pombe* mitotic and meiotic spindle assembly, and elongation

Ana Loncar, Sergio A. Rincon, Manuel Lera Ramirez, Anne Paoletti and Phong T. Tran
DOI: 10.1242/jcs.240234

Editor: David Glover

Review timeline
Original submission: 7 October 2019
Editorial decision: 9 December 2019
First revision received: 19 February 2020
Editorial decision: 26 March 2020
Second revision received: 31 March 2020
Accepted: 6 April 2020

Original submission

First decision letter

MS ID#: JOCES/2019/240234

MS TITLE: Kinesin-14s and microtubule dynamics define distinct mitotic and meiotic spindle assembly and elongation.

AUTHORS: Ana Loncar, Sergio Rincon, Manuel Lera Ramirez, Anne Paoletti, and Phong Tran
ARTICLE TYPE: Research Article

We have now reached a decision on the above manuscript.

To see the reviewers' reports and a copy of this decision letter, please go to: https://submit-jcs.biologists.org and click on the 'Manuscripts with Decisions' queue in the Author Area. (Corresponding author only has access to reviews.)

As you will see, the reviewers raise a number of substantial criticisms that prevent me from accepting the paper at this stage. They suggest, however, that a revised version might prove acceptable, if you can address their concerns. If you think that you can deal satisfactorily with the criticisms on revision, I would be pleased to see a revised manuscript. We would then return it to the reviewers.

Please ensure that you clearly highlight all changes made in the revised manuscript. Please avoid using 'Tracked changes' in Word files as these are lost in PDF conversion.

I should be grateful if you would also provide a point-by-point response detailing how you have dealt with the points raised by the reviewers in the 'Response to Reviewers' box. Please attend to all of the reviewers' comments. If you do not agree with any of their criticisms or suggestions please explain clearly why this is so.

© 2020. Published by The Company of Biologists under the terms of the Creative Commons Attribution License (https://creativecommons.org/licenses/by/4.0/).

Reviewer 1

Advance summary and potential significance to field

This study by Loncar et al. examines differences in the assembly and elongation dynamics of mitotic versus meiotic spindles. The study leverages the fission yeast model cell, which allows the authors to readily and directly compare components and characteristics of the two spindle types. Overall, compared to mitotic spindles, there is a lack of information regarding the molecular function of meiotic spindles and specifically how they differ from mitotic spindles. This paper is a solid advance in filling that gap. Interestingly, the authors find key differences between spindles in mitosis and meiosis I. They then show that these differences can be related to key spindle factors and the central concept of force balance within the spindles. The experiments and data analysis are well performed and of high quality, and the interpretations are sound and supported by the data. This study represents a significant advance that will facilitate more advanced studies into meiotic spindle functions.

Comments for the author

I have a number of relatively minor concerns.

Figs 3A and 4B, there is an overlay picture of red and green, and then one image of a single channel. Presumably the tagged motor is displayed as the single channel, but please specify on the figure and/or legend which single channel is displayed.

Fig 3C and D, the color of the short lines in the keys used to demark which color is used for each spindle type in the panel is difficult to discern because the lines are so thin. This may be improved with a thicker line in the key.

Fig 4C, the profile of Klp2 on the two spindle classes appears to have a fundamentally different shape, i.e. two peaks on the ends in MI spindles versus a single peak in the center of mitotic spindles. Is this difference significant and/or an artifact of the differences in spindle lengths? How could this difference impact the authors? interpretations of Klp2 localization, force balance and spindle function?

Fig 4E, it is difficult to interpret the degree to which the three distinct phases of spindle dynamics are rescued by klp2 deletion. This is in part because the red traces are heavily obscured by the scattered green traces. This may be improved by drawing the red on top of the green and/or increasing graph size, or perhaps graphing them separately. Similarly, line 255 refers to preanaphase spindle length being partially restored, yet this interpretation is not clear in the presentation of data in Fig 4F.

In Figure 5D the lines in the legend, which demark the colors used for the lines in the graph, are difficult to see clearly due to their thin width. This may benefit from making these legend symbols wider so that the difference in color is more obvious.

In Supp Fig 3C, due to the size of the graph it is difficult to see the profile of the red line underneath the green traces.

The title for Table 2 would benefit from specifying whether the microtubules measured and reported are spindle or astral microtubules.

Supp Fig 5D, is presented to support the idea that microtubules pivot around spindle poles more in meiotic compared to mitotic cells. This is difficult to discern and evaluate from only these images. In these images the spindles appear to be of different sizes and time points are up to 5 min apart. Could the authors more clearly quantitate and/or present this observation?

Line 228, it may be helpful to include the % of four spores to which the mutant is being compared to, e.g. 44% vs. what in cut7-delta pkl1-delta cells.

© 2020. Published by The Company of Biologists under the terms of the Creative Commons Attribution License (https://creativecommons.org/licenses/by/4.0/).

The paragraph beginning on line 194 has an unclear transition. The second sentence states that Cut7 is localized as previously reported (presumably for mitosis, but it is not clear whether it refers to both mitosis and meiosis). The third sentence then says 'Similarly,' Pkl1 is unchanged between the two spindle types. The second sentence did not make clear there is no change in Cut7 between the two.

In general, it felt like there were some places in which an article, e.g. 'the', would be beneficial. For example, line 226 "deleted motors and MAPs in the cut7-delta pkl1-delta background".

Cortes et al, 2018 is cited on line 311 but appears not to be listed in the references. However, it is present but formatted differently (thus appearing under the G's and not C's).

Reviewer 2

Advance summary and potential significance to field

This paper describes a detailed comparison of spindle structure and kinematics in fission yeast cells that are undergoing vegetative mitotic chromosome segregation vs. the meiotic divisions that follow conjugation of two mating types. The descriptions are based on high quality fluorescence imaging of proteins that have been expressed as chimeras with fluorescent proteins of different colors. Spindle sizes and the speeds of spindle growth are compared in useful ways. These parameters are also examined in cells that have been deleted for the gene encoding the cell’s one kinesin 5, cut7 as well as one or both of the cell’s kinesin 14s, allowing useful mechanistic statements about the roles of these motors and microtubule dynamics in different aspects of spindle structure and function. The work is well conducted and provides interesting new information about spindle function in these different stages of vegetative and sexual reproduction. Importantly, deletion of one kinesin 14 does rescue deletion of the essential kinesin 5 for vegetative growth, but not for meiosis. These results are then correlated with differences in ratios of the amounts of the two motors associated with the spindle. Deletion of the second kinesin 14 helps the situation, and modification of microtubule dynamics by a tubulin-binding drug, helps more, contributing evidence to support the widely held view that spindles function as a result of the competing action of several force generating systems. As such, the work is valuable and should be published. However, the paper could be improved in several ways, suggesting that some modifications are appropriate. Judicious cutting of the introduction and discussion would be valuable, since the former includes an almost text-book description of meiosis and the latter includes extensive repeats of information that was just stated in the Results section. However, the paper lacks essential information about the behavior of cells carrying these motor-fluorescent protein chimeras. This information is important for a confident interpretation of the morphological information presented. These details will help a careful reader to relate the descriptions given with the biology under consideration.

Comments for the author

Abstract: ln 36 “Consequently” is a term that makes sense only within the context of the model that kinesin 5s and 14s antagonize one another in spindle function. Thus, it expresses the author’s bias that their data should be interpreted this way. This model bias is found throughout the paper, and it gives the reader a sense that data were collected to prove a point rather than to answer a question. Changing that tone throughout the paper would make it a more professional piece of work.
Ln51 Pombe is not unique in its suitability for this kind of observation. Many other fungi could also be used, as well as organisms like C. elegans, where comparable work is ongoing.
Ln58 This reviewer is unhappy about the use of the term “prophase” for the early stages of spindle formation. In the classic literature, prophase has been defined as the time of chromosome condensation (which is not observed here). The time when chromosomes first engage with the spindle and take on the order characteristic of metaphase is called “prometatphase”. I realize that in budding yeast this kind of distinction is impossible, because chromosomes attach to the spindle poles and then spindle microtubules during S-phase, but in pombe, a more traditional use of the mitotic phases is perfectly straightforward. I believe it should be adopted here.
Ln99 “We fist show…” is ambiguous. Are the authors referring to this paper or other work?

© 2020. Published by The Company of Biologists under the terms of the Creative Commons Attribution License (https://creativecommons.org/licenses/by/4.0/).

Ln112 Descriptions of spindle growth rate are described as spindle dynamics. Although this is true in a sense, the word dynamics has commonly been used in mitosis to refer to the rates of growth, shortening, and turnover of the component microtubules. From a physicist's point of view, descriptions of the rates of movement are kinematics, not dynamics. Since this paper goes on at a later time to talk about microtubule growth rates, it might be clearer to your readers to use the kinematics term for what it is and reserve dynamics for the polymers.
The prose style in the whole paper but especially in the results section is quite luxurious. Phrases like "We noticed…" (Ln 123) "In order to understand, we decided to explore…" (Ln 135) add words with no real meaning. Of course you observed, noticed, or saw, because if you hadn't you would be able to say anything. Such phrases can almost always be cut.
Ln 198, 230, etc. These sections describe observations on the relative amounts of different motor enzymes on meiotic and mitotic spindles, interesting work. The strain table indicates that these strains were made for this study, but key aspects of strain behavior are not described, making it hard to interpret the results described. Was the locus of integration checked, and was the endogenous, wild type allele replaced? Did the addition of the fluorescent protein to the motor, which is of course a mutation, have a growth or structural phenotype? Unless this work is done and documented, the validity of the fluorescence intensity measurements is of questionable value. I strongly recommend that this information be added in supplementary figures, so the reader can believe that the observed ratios of amounts can be interpreted in the context of a wild or mutant phenotype.
Ln242 Please state in the text the numbers of cells examined, both for this and elsewhere for comparable experiments.
Ln269 The text confuses a microtubule catastrophe (the transition from growth to shortening) with the process of rapid shortening.

<u>Reviewer 3</u>

Advance summary and potential significance to field

In this study, Loncar et al, find and explore differences in Spindle dynamics between mitosis and meiosis. This is an interesting finding and understanding the balances of forces necessary for proper spindle assembly and chromosome segregation in both mitosis and meiosis is of fundamental importance in cell biology.

Comments for the author

In this manuscript, Loncar et al, by using the cut7Dpkl1D double mutant explore the differences in spindle behavior and dynamics between mitosis and meiosis. In proliferating fission yeast cells inactivation of kinesin 5 (cut7) leads to monopolar spindles however spindle bipolarity is restored in cells defective simultaneously for kinesin 5 cut7and kinesin-14 Pkl1.
In meiosis however, Loncar et al show in this manuscript that cut7 cannot be suppressed by deletion of pkl1 which suggests that mitotic and MI spindles may have fundamental differences in composition or dynamics affecting overall balance of forces.
The authors show that mitosis and meiosis spindles show different lengths and dynamics. Based on these differences they suggest quantitative or qualitative changes in spindle components between mitosis and MI. They find that while total amounts of cut7 scales up with MT intensity from mitosis to MI total Pkl1 remains constant. This would result in an increase in net outward forces produced by MI spindle relative to mitosis and explains the phenotype of lack of suppression of cut7D by klp1D during MI. They reason that other factor might compensate for the increase in cut7-dependent forces (or the lower levels of Pkl1 relative to MT intensity) in MI spindle in order to maintain the balances of forces. The authors then perform a systematic search of MAPs whose deletion restored spindle formation, chromosome segregation and the formation of 4 spores in the cut7 klp1 double mutant during meiosis, and found Klp2 deletion as the only suppressor.
They convincingly show that klp2 deletion rescues cut7 pkl11 independent spindle formation in MI. The analysis of klp2 localization showed an increase in the total amount of Klp2 in MI compared to mitosis. Therefore they propose Klp2 as a new player in counteracting the Cut7-dependent outward pushing forces during MI.

© 2020. Published by The Company of Biologists under the terms of the Creative Commons Attribution License (https://creativecommons.org/licenses/by/4.0/).

Finally, they explore MT dynamics as another possible factor influencing the balance of forces produced by the spindle. They find that MTs are more dynamic during MI than during Mitosis. They test whether MT dynamics contribute to counteract Cut7 dependent outward forces during MI by altering MT dynamics with low doses of MBC. They found that whereas in wt cells low doses of MBC results in a slightly lower frequency of zygotes with 4 spores, in cut7Dpkl1D zygotes the same low doses of MBC, results in a higher frequency of zygotes producing 4 spores and thus suppresses cut7Dpkl1D phenotype.

In general I have enjoyed reading this manuscript. The data is clear and well presented and understanding the balances of forces necessary for proper spindle assembly and chromosome segregation is an important problem in cell biology.

I have however some concerns and comments listed below.

Major points

Figure S1E is hard to understand. The idea behind this observation is important for the message of this work as it suggests that pkl1 deleted cells can not fully compensate the absence of cut7 resulting in unbalanced spindle forces. I suggest this data is presented more clearly. As Fig S1E is an inset of a bigger picture it would be better to show the whole cell / nucleus and indicate where the inset was taken. That would facilitate the understanding for non-S. *pombe* readers and the data would be more convincing for the rest. Additionally, the frequency of the different phenotypes might also be shown in the figure (here and in Figure 2C).

Judged by cdc13 levels, the author shows that in spite of a failure in MI spindle formation, cells initiate MII (cdc13 level increased again after its degradation at the end of MI). The authors concluded that progression through meiotic stages goes unchecked regardless of spindle or chromosome defects. In order to say that, authors should show timing of cyclin B degradation during MI and MII relative to wild type. It is possible that defects in spindle formation and chromosome segregation are indeed detected by the SAC and the progression through meiosis delayed to certain extent, however, as all the checkpoints, its leakage leads to cell cycle progression. Authors should quantitate cycles of cyclin B degradation during meiosis in wt and cut7 klp1D to check whether defects of spindle formation and chromosome segregation during MI and MII are indeed delaying meiotic progression, or if as authors suggest, these defects go unnoticed, which would be surprising.

The author claim that klp2 localized more strongly to spindle in prometaphase of MI relative to mitosis, which would be consistent with their hypothesis, however whereas the increased levels of Cut7 at MI (Figure 3A) is clearly seen, the increased levels of Klp2 is difficult to infer from Figure 4B. The author should show a more representative image of Klp2 during Mitosis and Meiosis.

Page 3 line 55: “Fission yeast can complete mitosis and meiosis in the same environment”. Did they analyze mitotic cells in rich media or did they analyze those cells in ME (mating media) that go through the last mitosis prior to sexual differentiation?
If this is the case, mitosis in minimal media might not follow exactly the same dynamics than in rich media due to nutrient starvation sensing…I suggest comparing mitotic parameters in rich and ME media to make sure that they behave similarly and perhaps include this information in a supplemental figure.

Minor point

As the authors have already shown changes in spindle dynamics (table I and Fig1), in line 158 authors should say, we decided to explore spindle dynamics in this mutant.

Timing is not depicted in any of the figures.

-Page 7 line 165. The expression “we saw an increase in SPB number” might not be clear for non-pombe readers. This might be clarified

© 2020. Published by The Company of Biologists under the terms of the Creative Commons Attribution License (https://creativecommons.org/licenses/by/4.0/).

First revision

Author response to reviewers' comments

Response to Reviewers

We thank the Reviewers for their time and effort in reading our manuscript. The general enthusiasm from the Reviewers for the potential significance of this work in comparing mitotic and meiotic spindles is much appreciated. In the revision, we made additional experiments/analysis, modified some of the figures, and clarified the text. Textual changes are indicated in yellow. We have addressed all of the suggestions from the Reviewers. Below are point-by-point answers to the questions raised.

Reviewer 1 Advance Summary and Potential Significance to Field...

This study by Loncar et al. examines differences in the assembly and elongation dynamics of mitotic versus meiotic spindles. The study leverages the fission yeast model cell, which allows the authors to readily and directly compare components and characteristics of the two spindle types. Overall, compared to mitotic spindles, there is a lack of information regarding the molecular function of meiotic spindles and specifically how they differ from mitotic spindles. This paper is a solid advance in filling that gap. Interestingly, the authors find key differences between spindles in mitosis and meiosis I. They then show that these differences can be related to key spindle factors and the central concept of force balance within the spindles. The experiments and data analysis are well performed and of high quality, and the interpretations are sound and supported by the data. This study represents a significant advance that will facilitate more advanced studies into meiotic spindle functions.

Reviewer 1 Comments for the Author...

I have a number of relatively minor concerns.

Figs 3A and 4B, there is an overlay picture of red and green, and then one image of a single channel. Presumably the tagged motor is displayed as the single channel, but please specify on the figure and/or legend which single channel is displayed.

Figure 4B is now Figure 4D. Indeed, the Reviewer presumed correctly. We have added the necessary information to the figure (and similar figures elsewhere when applicable).

Fig 3C and D, the color of the short lines in the keys used to demark which color is used for each spindle type in the panel is difficult to discern because the lines are so thin. This may be improved with a thicker line in the key.

We have increased the size of the lines in the figure here (and elsewhere when applicable).

Fig 4C, the profile of Klp2 on the two spindle classes appears to have a fundamentally different shape, i.e. two peaks on the ends in MI spindles versus a single peak in the center of mitotic spindles. Is this difference significant and/or an artifact of the differences in spindle lengths? How could this difference impact the authors' interpretations of Klp2 localization, force balance and spindle function?

Figure 4C is now Figure 4E. We think the signal is a true representation of Klp2-GFP localization in mitotic versus meiotic I spindles. We clarify our view with new text in the result and discussions.

Fig 4E, it is difficult to interpret the degree to which the three distinct phases of spindle dynamics are rescued by klp2 deletion. This is in part because the red traces are heavily obscured by the scattered green traces. This may be improved by drawing the red on top of the green and/or increasing graph size, or perhaps graphing them separately. Similarly, line 255 refers to pre-anaphase spindle length being partially restored, yet this interpretation is not clear in the presentation of data in Fig 4F.

© 2020. Published by The Company of Biologists under the terms of the Creative Commons Attribution License (https://creativecommons.org/licenses/by/4.0/).

Figure 4F is now Figure 4C.We have now increased the graph size, plotted the red lines on top of the green lines, and added two dotted lines representing the average pre-phase III spindle length in *cut7Δpkl1Δ* and *cut7Δpkl1Δklp2Δ* cells. We have also included an additional box-and-whiskers plot comparing of the pre-phase III spindle length in MI of the two mutants.

In Figure 5D the lines in the legend, which demark the colors used for the lines in the graph, are difficult to see clearly due to their thin width. This may benefit from making these legend symbols wider so that the difference in color is more obvious.

We have increased the size of the lines in the figure here (and elsewhere when applicable).

In Supp Fig 3C, due to the size of the graph it is difficult to see the profile of the red line underneath the green traces.

Supplemental Fig. 3C is now Supplemental Fig. 4C. We have increased the size of the lines in the figure here (and elsewhere when applicable).

The title for Table 2 would benefit from specifying whether the microtubules measured and reported are spindle or astral microtubules.

The MTs measured in this experiments are neither astral nor spindle MTs. The spindle contains many MTs, and therefore we could not measure individual MT dynamics inside a spindle. Instead, we cytoplasmic MTs prior to mitosis (IP) and during the pause between MI and MII (interkinesis or IK). These MTs are individual bundle of MTs, acting as proxy of MT dynamics during mitosis and meiosis. We have now explicitly stated this in the text.

Supp Fig 5D, is presented to support the idea that microtubules pivot around spindle poles more in meiotic compared to mitotic cells. This is difficult to discern and evaluate from only these images. In these images the spindles appear to be of different sizes and time points are up to 5 min apart. Could the authors more clearly quantitate and/or present this observation?

Supplemental Fig. 5D is now Supplemental Fig. 5F. We have created a new figure comparing the first 200 seconds of spindle dynamics at phase I onset of mitosis and MI.

Line 228, it may be helpful to include the % of four spores to which the mutant is being compared to, e.g. 44% vs. what in cut7-delta pkl1-delta cells.

Yes, we have now clarify this in the text.

The paragraph beginning on line 194 has an unclear transition. The second sentence states that Cut7 is localized as previously reported (presumably for mitosis, but it is not clear whether it refers to both mitosis and meiosis). The third sentence then says 'Similarly,' Pkl1 is unchanged between the two spindle types. The second sentence did not make clear there is no change in Cut7 between the two.

We have clarified that Cut7-GFP localized similarly on both mitotic and meiotic spindles.

In general, it felt like there were some places in which an article, e.g. 'the', would be beneficial. For example, line 226 "deleted motors and MAPs in the cut7-delta pkl1-delta background".

Yes, we have added numerous “the” throughout the text.

Cortes et al, 2018 is cited on line 311 but appears not to be listed in the references. However, it is present but formatted differently (thus appearing under the G's and not C's).

We have now properly formatted all the references, including adding a new reference.

Reviewer 2 Advance Summary and Potential Significance to Field...

This paper describes a detailed comparison of spindle structure and kinematics in fission yeast cells

© 2020. Published by The Company of Biologists under the terms of the Creative Commons Attribution License (https://creativecommons.org/licenses/by/4.0/).

that are undergoing vegetative mitotic chromosome segregation vs. the meiotic divisions that follow conjugation of two mating types. The descriptions are based on high quality fluorescence imaging of proteins that have been expressed as chimeras with fluorescent proteins of different colors. Spindle sizes and the speeds of spindle growth are compared in useful ways. These parameters are also examined in cells that have been deleted for the gene encoding the cell's one kinesin 5, cut7 as well as one or both of the cell's kinesin 14s, allowing useful mechanistic statements about the roles of these motors and microtubule dynamics in different aspects of spindle structure and function. The work is well conducted and provides interesting new information about spindle function in these different stages of vegetative and sexual reproduction. Importantly, deletion of one kinesin 14 does rescue deletion of the essential kinesin 5 for vegetative growth, but not for meiosis. These results are then correlated with differences in ratios of the amounts of the two motors associated with the spindle. Deletion of the second kinesin 14 helps the situation, and modification of microtubule dynamics by a tubulin-binding drug, helps more, contributing evidence to support the widely held view that spindles function as a result of the competing action of several force generating systems. As such, the work is valuable and should be published.

However, the paper could be improved in several ways, suggesting that some modifications are appropriate. Judicious cutting of the introduction and discussion would be valuable, since the former includes an almost text-book description of meiosis and the latter includes extensive repeats of information that was just stated in the Results section. However, the paper lacks essential information about the behavior of cells carrying these motor-fluorescent protein chimeras. This information is important for a confident interpretation of the morphological information presented. These details will help a careful reader to relate the descriptions given with the biology under consideration.

We have made effort to cut all repeats of information, making the introduction, result, and discussion sections slightly shorter.

In addition, we have added new figures/text describing the behaviour of Cut7, Pkl1, and Klp2 tagged with GFP. The new data indicate that GFP-tagging does have a small effect on these motors, making them slightly less functional compared to their non- tagged version. For example, some phases of spindle dynamics are slightly prolonged. However, in term of cell growth and proliferation, spore number, and bipolar spindle formation, the GFP-tagged strains are indistinguishable from the non-tagged strains.

Reviewer 2 Comments for the Author...

Abstract: ln 36 "Consequently" is a term that makes sense only within the context of the model that kinesin 5s and 14s antagonize one another in spindle function. Thus, it expresses the author's bias that their data should be interpreted this way. This model bias is found throughout the paper, and it gives the reader a sense that data were collected to prove a point rather than to answer a question. Changing that tone throughout the paper would make it a more professional piece of work.

We have made effort throughout the text to tone down our wording to prevent bias.

Ln51 Pombe is not unique in its suitability for this kind of observation. Many other fungi could also be used, as well as organisms like C. elegans, where comparable work is ongoing.

We removed the word "unique". Clearly, comparative mitosis and meiosis can be studied in many organisms. However, we emphasize that our current work using fission yeast may be the first to character both process at the same time, in the same imaging condition.

Ln58 This reviewer is unhappy about the use of the term "prophase" for the early stages of spindle formation. In the classic literature, prophase has been defined as the time of chromosome condensation (which is not observed here). The time when chromosomes first engage with the spindle and take on the order characteristic of metaphase is called "prometatphase". I realize that in budding yeast this kind of distinction is impossible, because chromosomes attach to the spindle poles and then spindle microtubules during S-phase, but in pombe, a more traditional use of the mitotic

© 2020. Published by The Company of Biologists under the terms of the Creative Commons Attribution License (https://creativecommons.org/licenses/by/4.0/).

phases is perfectly straightforward. I believe it should be adopted here.

We agree with the Reviewer that in fission yeast, the precise definition of prophase may not be evident by imaging the microtubule spindle alone. We have now defined spindle assembly and elongation strictly in term of the three-phase: phase I=prophase/prometaphase, phase II=metaphase/anaphaseA and phase III=anaphaseB.

Ln99 “We first show…” is ambiguous. Are the authors referring to this paper or other work?

We clarified the text to refer to the current work.

Ln112 Descriptions of spindle growth rate are described as spindle dynamics. Although this is true in a sense, the word dynamics has commonly been used in mitosis to refer to the rates of growth, shortening, and turnover of the component microtubules. From a physicist’s point of view, descriptions of the rates of movement are kinematics, not dynamics. Since this paper goes on at a later time to talk about microtubule growth rates, it might be clearer to your readers to use the kinematics term for what it is and reserve dynamics for the polymers.

We agree with the Reviewer that “spindle dynamics” is distinct from “spindle kinematics”. However, historically, “spindle dynamics” is frequently used to describe length changes in the spindle. Outside of the McIntosh and Scholey labs, we did not encounter “spindle kinematics” elsewhere. Thus, we would like to keep this historical precedent.

The prose style in the whole paper but especially in the results section is quite luxurious. Phrases like “We noticed…” (Ln 123) “In order to understand, we decided to explore…” (Ln 135) add words with no real meaning. Of course you observed, noticed, or saw, because if you hadn’t you would be able to say anything. Such phrases can almost always be cut.

We have removed, where applicable, the extraneous wordings.

Ln 198, 230, etc. These sections describe observations on the relative amounts of different motor enzymes on meiotic and mitotic spindles, interesting work. The strain table indicates that these strains were made for this study, but key aspects of strain behavior are not described, making it hard to interpret the results described. Was the locus of integration checked, and was the endogenous, wild type allele replaced? Did the addition of the fluorescent protein to the motor, which is of course a mutation, have a growth or structural phenotype? Unless this work is done and documented, the validity of the fluorescence intensity measurements is of questionable value. I strongly recommend that this information be added in supplementary figures, so the reader can believe that the observed ratios of amounts can be interpreted in the context of a wild or mutant phenotype.

The GFP-tagging of the motors Cut7, Pkl1, and Klp2 was performed at their C- terminus, and at their endogenous locus. This enabled the respective endogenous promoter to control the protein expression level. Tagged strains were checked by PCR. We have now clarified this in the Methods section.

As stated above, the GFP-tagged motors are not completely wild-type. However, in term of bipolar spindle formation, spore number, and rate of cell growth/proliferation, the GFP-tagged behave similar to non-tagged wild-type strains. Thus, our results likely are not an artefact of GFP-tagging. The this revision, we have added extensively to the result section, including new supplemental figures 3 and 4, to describe the behaviour of the GFP-tagged strains compared to non-tagged strains.

Ln242 Please state in the text the numbers of cells examined, both for this and elsewhere for comparable experiments.

To prevent numerous repetitions, we opted to include the n-values for each experiments in the figures and figure legends.

Ln269 The text confuses a microtubule catastrophe (the transition from growth to shortening) with the process of rapid shortening.

© 2020. Published by The Company of Biologists under the terms of the Creative Commons Attribution License (https://creativecommons.org/licenses/by/4.0/).

We have clarified the text.

Reviewer 3 Advance Summary and Potential Significance to Field...

In this study, Loncar et al, find and explore differences in Spindle dynamics between mitosis and meiosis. This is an interesting finding and understanding the balances of forces necessary for proper spindle assembly and chromosome segregation in both mitosis and meiosis is of fundamental importance in cell biology.

Reviewer 3 Comments for the Author...

In this manuscript, Loncar et al, by using the cut7Dpkl1D double mutant explore the differences in spindle behavior and dynamics between mitosis and meiosis. In proliferating fission yeast cells inactivation of kinesin 5 (cut7) leads to monopolar spindles however spindle bipolarity is restored in cells defective simultaneously for kinesin 5 cut7and kinesin-14 Pkl1.

In meiosis however, Loncar et al show in this manuscript that cut7 cannot be suppressed by deletion of pkl1 which suggests that mitotic and MI spindles may have fundamental differences in composition or dynamics affecting overall balance of forces.

The authors show that mitosis and meiosis spindles show different lengths and dynamics. Based on these differences they suggest quantitative or qualitative changes in spindle components between mitosis and MI. They find that while total amounts of cut7 scales up with MT intensity from mitosis to MI total Pkl1 remains constant. This would result in an increase in net outward forces produced by MI spindle relative to mitosis and explains the phenotype of lack of suppression of cut7D by klp1D during MI. They reason that other factor might compensate for the increase in cut7-dependent forces (or the lower levels of Pkl1 relative to MT intensity) in MI spindle in order to maintain the balances of forces. The authors then perform a systematic search of MAPs whose deletion restored spindle formation, chromosome segregation and the formation of 4 spores in the cut7 klp1 double mutant during meiosis, and found Klp2 deletion as the only suppressor. They convincingly show that klp2 deletion rescues cut7 pkl11 independent spindle formation in MI. The analysis of klp2 localization showed an increase in the total amount of Klp2 in MI compared to mitosis. Therefore they propose Klp2 as a new player in counteracting the Cut7-dependent outward pushing forces during MI.

Finally, they explore MT dynamics as another possible factor influencing the balance of forces produced by the spindle. They find that MTs are more dynamic during MI than during Mitosis. They test whether MT dynamics contribute to counteract Cut7 dependent outward forces during MI by altering MT dynamics with low doses of MBC. They found that whereas in wt cells low doses of MBC results in a slightly lower frequency of zygotes with 4 spores, in cut7Dpkl1D zygotes the same low doses of MBC, results in a higher frequency of zygotes producing 4 spores and thus suppresses cut7Dpkl1D phenotype.

In general I have enjoyed reading this manuscript. The data is clear and well presented and understanding the balances of forces necessary for proper spindle assembly and chromosome segregation is an important problem in cell biology.

I have however some concerns and comments listed below.

Major points

Figure S1E is hard to understand. The idea behind this observation is important for the message of this work as it suggests that pkl1 deleted cells can not fully compensate the absence of cut7 resulting in unbalanced spindle forces. I suggest this data is presented more clearly. As Fig S1E is an inset of a bigger picture it would be better to show the whole cell / nucleus and indicate where the inset was taken. That would facilitate the understanding for non-S. pombe readers and the data would be more convincing for the rest. Additionally, the frequency of the different phenotypes might also be shown in the figure (here and in Figure 2C).

© 2020. Published by The Company of Biologists under the terms of the Creative Commons Attribution License (https://creativecommons.org/licenses/by/4.0/).

Supplemental Fig. 1E is now Supplemental Fig. 2G. The image of the whole zygote is now clearly shown, with magnified inset.

Figure 2C phenotype percentages have been added to the figure and the figure legend.

Judged by cdc13 levels, the author shows that in spite of a failure in MI spindle formation, cells initiate MII (cdc13 level increased again after its degradation at the end of MI). The authors concluded that progression through meiotic stages goes unchecked regardless of spindle or chromosome defects. In order to say that, authors should show timing of cyclin B degradation during MI and MII relative to wild type. It is possible that defects in spindle formation and chromosome segregation are indeed detected by the SAC and the progression through meiosis delayed to certain extent, however, as all the checkpoints, its leakage leads to cell cycle progression. Authors should quantitate cycles of cyclin B degradation during meiosis in wt and cut7 klp1D to check whether defects of spindle formation and chromosome segregation during MI and MII are indeed delaying meiotic progression, or if as authors suggest, these defects go unnoticed, which would be surprising.

Supplemental Fig. 2F was added to show Cdc13-GFP degradation timing in MI of *wt* and *cut7Δpkl1Δ* zygotes. We have amended the text to state that the SAC was active and that phase III onset was delayed in MI of the mutant zygotes when compared to the *wt*.

The author claim that klp2 localized more strongly to spindle in prometaphase of MI relative to mitosis, which would be consistent with their hypothesis, however whereas the increased levels of Cut7 at MI (Figure 3A) is clearly seen, the increased levels of Klp2 is difficult to infer from Figure 4B. The author should show a more representative image of Klp2 during Mitosis and Meiosis.

Figure 4B is now Figure 4D. We have found better representative cells for Figure 4D. Nevertheless, the Klp2-GFP signal is inherently weak.

Page 3 line 55: “Fission yeast can complete mitosis and meiosis in the same environment”. Did they analyze mitotic cells in rich media or did they analyze those cells in ME (mating media) that go through the last mitosis prior to sexual differentiation?

If this is the case, mitosis in minimal media might not follow exactly the same dynamics than in rich media due to nutrient starvation sensing…I suggest comparing mitotic parameters in rich and ME media to make sure that they behave similarly and perhaps include this information in a supplemental figure.

We have analysed spindle dynamics in mitotic cells in rich medium (YE5S) and mating medium (ME). The data is shown in Supplemental Fig. 1A. We found mitotic spindle dynamics to be comparable between cells grown in YE5S or ME. However, anaphase B (phase III) of cells in ME showed a slight decrease in spindle elongation velocity, resulting in a modest 10% increase in total mitosis duration.

Minor point

As the authors have already shown changes in spindle dynamics (table I and Fig1), in line 158 authors should say, we decided to explore spindle dynamics in this mutant.

We have modified the text accordingly.

Timing is not depicted in any of the figures.

We have added time stamps, where appropriate, on all figures.

Page 7 line 165. The expression “we saw an increase in SPB number” might not be clear for non-pombe readers. This might be clarified.

We have clarified the text.

In conclusion, through additional experiments, new figures and modified text, we have addressed all

© 2020. Published by The Company of Biologists under the terms of the Creative Commons Attribution License (https://creativecommons.org/licenses/by/4.0/).

comments from the Reviewers. We appreciate the previous opinion that this work is of importance in cell biology. We hope that the revised manuscript now merits acceptance for publication.

Ana Loncar & Phong Tran.

Second decision letter

MS ID#: JOCES/2019/240234

MS TITLE: Kinesin-14s and microtubule dynamics define distinct mitotic and meiotic spindle assembly and elongation.

AUTHORS: Ana Loncar, Sergio Rincon, Manuel Lera Ramirez, Anne Paoletti, and Phong Tran
ARTICLE TYPE: Research Article

We have now reached a decision on the above manuscript.

To see the reviewers' reports and a copy of this decision letter, please go to: https://submit-jcs.biologists.org and click on the 'Manuscripts with Decisions' queue in the Author Area. (Corresponding author only has access to reviews.)

As you will see, the reviewers gave favourable reports but raised some critical points that will require amendments to your manuscript. I hope that you will be able to carry these out, because I would like to be able to accept your paper.

We are aware that you may currently be unable to access the lab to undertake experimental revisions. If it would be helpful, we encourage you to contact us to discuss your revision in greater detail. Please send us a point-by-point response indicating where you are able to address concerns raised (either experimentally or by changes to the text) and where you will not be able to do so within the normal timeframe of a revision. We will then provide further guidance. Please also note that we are happy to extend revision timeframes as necessary.

Please ensure that you clearly highlight all changes made in the revised manuscript. Please avoid using 'Tracked changes' in Word files as these are lost in PDF conversion.

I should be grateful if you would also provide a point-by-point response detailing how you have dealt with the points raised by the reviewers in the 'Response to Reviewers' box. Please attend to all of the reviewers' comments. If you do not agree with any of their criticisms or suggestions please explain clearly why this is so.

Reviewer 1

Advance summary and potential significance to field

This Reviewer believes the authors Loncar et al. have done an overall nice job of addressing the Reviewers' comments. While the authors have performed several new experiments and added data to strengthen the manuscript, the resulting product appears rushed and lacks the quality of a finished manuscript. In its final form, this paper will be a valuable advance in filling the gap in knowledge between mitotic and meiotic spindle function. It will lay a foundation from which to facilitate future studies into meiotic spindle functions.

Comments for the author

Overall, the authors have undertaken meaningful steps to address Reviewers' comments. However, the revised manuscript contains numerous errors that must be corrected and carefully proofread

© 2020. Published by The Company of Biologists under the terms of the Creative Commons Attribution License (https://creativecommons.org/licenses/by/4.0/).

before the manuscript is sufficient quality for publication. Although not exhaustive, the examples below are representative of things that should be carefully considered:

Figure S5, it is difficult to visualize the pivoting microtubules. In most cases they are visible when demarked by the yellow arrow and then appear absent (or uncertain) in the following panel. Can pivoting be distinguished from depolymerization? Are there more clear examples?

In new figure 4C, it is now difficult to discern the green lines underneath the red lines. So although the authors attempted to increase visibility by reversing the order it unfortunately did not significantly help this Reviewer visualize the traces. I will leave this up to the authors discretion, with the feedback that this figure may be difficult for readers to discern spindle behavior at lower lengths. Same holds true for other similar figures.

On page 7, the analysis revealed that cut7Δpkl1Δ zygotes formed monopolar protrusions with distal MT-plus ends (Fig. S2F). – should cite Fig. S2G

On page 11, linescan analysis of Klp2-GFP showed higher maximum intensity peaks in MI, indicating an increase in the total amount of Klp2-GFP in MI compared to mitosis (Fig. 4C). However, the normalized concentration of Klp2-GFP relative to spindle MTs remained unchanged between mitotic and MI (Fig. 4D). – should cite Fig. 4E and Fig. 4F.

Top of page 12: “meiotic IK MTs are more dynamics than mitotic IP MTs” should be “meiotic IK MTs are more dynamic than mitotic IP MTs”

Page 14, “by measuring the intensities in the same cell but in different type of cell division, the differences…” “by measuring the intensities in the same cell but in different types of cell division, the differences…”

Page 17, “some of the patch in water so the OD was 1.5.”. Please indicate what wavelength is referred to by OD.

Check manuscript for typos and accurate figure references.

Reviewer 2

Advance summary and potential significance to field

The paper provides a careful description of spindle behavior in fission yeast cells as they go through either mitosis or meiosis. Some genetic and pharmacological perturbations are used to assess the importance of specific motor enzymes and microtubule dynamics in these two division processes, and some differences are seen. Although the paper is largely descriptive and contains only a few truly novel observations, the work is well done and provides a clear and definitive description of the similarities and differences between these two division processes in an important experimental organism. As such, the paper will be widely read and sited, so publication is appropriate.

Comments for the author

The authors have done a good job of incorporating the suggestions from their previous review. Although they have preserved some of the wordings to which I previously took exception, this is their paper, and I think the current version is suitable for publication.

Reviewer 3

Advance summary and potential significance to field

In this manuscript, Loncar et al, by using the cut7Dpkl1D double mutant explore the differences in spindle behavior and dynamics between mitosis and meiosis. In proliferating fission yeast cells

© 2020. Published by The Company of Biologists under the terms of the Creative Commons Attribution License (https://creativecommons.org/licenses/by/4.0/).

inactivation of kinesin 5 (cut7) leads to monopolar spindles however spindle bipolarity is restored in cells defective simultaneously for kinesin 5 cut7 and kinesin-14 Pkl1.

In meiosis however, Loncar et al show in this manuscript that cut7 cannot be suppressed by deletion of pkl1 which suggests that mitotic and MI spindles may have fundamental differences in composition or dynamics affecting overall balance of forces.
The authors show that mitosis and meiosis spindles show different lengths and dynamics.
Based on these differences they suggest quantitative or qualitative changes in spindle components between mitosis and MI. They find that while total amounts of cut7 scales up with MT intensity from mitosis to MI total Pkl1 remains constant. This would result in an increase in net outward forces produced by MI spindle relative to mitosis and explains the phenotype of lack of suppression of cut7D by klp1D during MI. They reason that other factors might compensate for the increase in cut7-dependent forces (or the lower levels of Pkl1 relative to MT intensity) in MI spindle in order to maintain the balances of forces. The authors then perform a systematic search of MAPs whose deletion restored spindle formation, chromosome segregation and the formation of 4 spores in the cut7 klp1 double mutant during meiosis, and found Klp2 deletion as the only suppressor. They convincingly show that klp2 deletion rescues cut7 pkl11 independent spindle formation in MI. The analysis of klp2 localization showed an increase in the total amount of Klp2 in MI compared to mitosis. Therefore they propose Klp2 as a new player in counteracting the Cut7-dependent outward pushing forces during MI.

Finally, they explore MT dynamics as another possible factor influencing the balance of forces produced by the spindle. They find that MTs are more dynamic during MI than during Mitosis. They test whether MT dynamics contribute to counteract Cut7 dependent outward forces during MI by altering MT dynamics with low doses of MBC. They found that whereas in wt cells low doses of MBC results in a slightly lower frequency of zygotes with 4 spores, in cut7Dpkl1D zygotes the same low doses of MBC, results in a higher frequency of zygotes producing 4 spores and thus suppresses cut7Dpkl1D phenotype.

In general I have enjoyed reading this manuscript. The data is clear and well presented and understanding the balances of forces necessary for proper spindle assembly and chromosome segregation is an important problem in cell biology.

Comments for the author

The new version of the manuscript entitled "Kinesin-14s and microtubule dynamics define fission yeast mitotic and meiotic spindle assembly and elongation" by Loncar et al. has addressed all my comments. The manuscript has been improved significantly and I have no further concern.

Second revision

Author response to reviewers' comments

We thank the Reviewers for the second round of effort in reading our paper. In general, all three Reviewers have recommended publication. However, Reviewer 1 requested a thorough proofreading of the paper for grammatical and typographical errors, proper citations, and enhancement of some figures.

In this version, we have addressed all of the suggestions from Reviewer 1. Briefly, we have performed spellcheck for the paper, and added proper citations. Textual changes are highlighted yellow. Below is the point-by-point response:

Reviewer 1 Advance Summary and Potential Significance to Field:

This Reviewer believes the authors Loncar et al. have done an overall nice job of addressing the Reviewers’ comments. While the authors have performed several new experiments and added data

© 2020. Published by The Company of Biologists under the terms of the Creative Commons Attribution License (https://creativecommons.org/licenses/by/4.0/).

to strengthen the manuscript, the resulting product appears rushed and lacks the quality of a finished manuscript. In its final form, this paper will be a valuable advance in filling the gap in knowledge between mitotic and meiotic spindle function. It will lay a foundation from which to facilitate future studies into meiotic spindle functions.

Reviewer 1 Comments for the Author:

Overall, the authors have undertaken meaningful steps to address Reviewers' comments. However, the revised manuscript contains numerous errors that must be corrected and carefully proofread before the manuscript is sufficient quality for publication. Although not exhaustive, the examples below are representative of things that should be carefully considered:

Figure S5, it is difficult to visualize the pivoting microtubules. In most cases they are visible when demarked by the yellow arrow and then appear absent (or uncertain) in the following panel. Can pivoting be distinguished from depolymerization? Are there more clear examples?

Figure S5F is a typical example of differences of microtubule dynamics between mitosis and meiosis. What is clear from the figure is that there are more microtubule "events" in meiosis compared to mitosis, over the same period of observation. We initially interpreted these events as microtubule pivoting. We have now revised our interpretation as potential microtubule pivoting or microtubule polymerization.

In new figure 4C, it is now difficult to discern the green lines underneath the red lines. So although the authors attempted to increase visibility by reversing the order it unfortunately did not significantly help this Reviewer visualize the traces. I will leave this up to the authors discretion, with the feedback that this figure may be difficult for readers to discern spindle behavior at lower lengths. Same holds true for other similar figures.

We first attempted to re-size Figure 4C (and other similar figures). However, overlapping data points/colors were always obscured regardless of size. We decided finally to leave the figures are they are. One positive point about these figures is that the overlap region signifies both populations having the same value. For our comparison, differences are more important than similarities. The figures show differences very clearly.

On page 7, the analysis revealed that cut7Δpkl1Δ zygotes formed monopolar protrusions with distal MT-plus ends (Fig. S2F). – should cite Fig. S2G.

This has been corrected.

On page 11, linescan analysis of Klp2-GFP showed higher maximum intensity peaks in MI, indicating an increase in the total amount of Klp2-GFP in MI compared to mitosis (Fig. 4C). However, the normalized concentration of Klp2-GFP relative to spindle MTs remained unchanged between mitotic and MI (Fig. 4D). – should cite Fig. 4E and Fig. 4F.

This has been corrected.

Top of page 12: "meiotic IK MTs are more dynamics than mitotic IP MTs" should be "meiotic IK MTs are more dynamic than mitotic IP MTs".

This has been corrected.

Page 14, "by measuring the intensities in the same cell but in different type of cell division, the differences…" should be "by measuring the intensities in the same cell but in different types of cell division, the differences…"

This has been corrected.

Page 17, "some of the patch in water so the OD was 1.5.". Please indicate what wavelength is referred to by OD.

© 2020. Published by The Company of Biologists under the terms of the Creative Commons Attribution License (https://creativecommons.org/licenses/by/4.0/).

The wavelength used was 600nm. This is now stated in the text.

Check manuscript for typos and accurate figure references.

We have performed a spellcheck and a check for proper figure citation.

Reviewer 2 Advance Summary and Potential Significance to Field:

The paper provides a careful description of spindle behavior in fission yeast cells as they go through either mitosis or meiosis. Some genetic and pharmacological perturbations are used to assess the importance of specific motor enzymes and microtubule dynamics in these two division processes, and some differences are seen. Although the paper is largely descriptive and contains only a few truly novel observations, the work is well done and provides a clear and definitive description of the similarities and differences between these two division processes in an important experimental organism. As such, the paper will be widely read and sited, so publication is appropriate.

Reviewer 2 Comments for the Author:

The authors have done a good job of incorporating the suggestions from their previous review. Although they have preserved some of the wordings to which I previously took exception, this is their paper, and I think the current version is suitable for publication.

We thank you for your positive review and recommendation.

Reviewer 3 Advance Summary and Potential Significance to Field:

In this manuscript, Loncar et al, by using the cut7Dpkl1D double mutant explore the differences in spindle behavior and dynamics between mitosis and meiosis. In proliferating fission yeast cells inactivation of kinesin 5 (cut7) leads to monopolar spindles however spindle bipolarity is restored in cells defective simultaneously for kinesin 5 cut7 and kinesin-14 Pkl1.

In meiosis however, Loncar et al show in this manuscript that cut7 cannot be suppressed by deletion of pkl1 which suggests that mitotic and MI spindles may have fundamental differences in composition or dynamics affecting overall balance of forces. The authors show that mitosis and meiosis spindles show different lengths and dynamics. Based on these differences they suggest quantitative or qualitative changes in spindle components between mitosis and MI. They find that while total amounts of cut7 scales up with MT intensity from mitosis to MI total Pkl1 remains constant. This would result in an increase in net outward forces produced by MI spindle relative to mitosis and explains the phenotype of lack of suppression of cut7D by klp1D during MI. They reason that other factors might compensate for the increase in cut7-dependent forces (or the lower levels of Pkl1 relative to MT intensity) in MI spindle in order to maintain the balances of forces. The authors then perform a systematic search of MAPs whose deletion restored spindle formation, chromosome segregation and the formation of 4 spores in the cut7 klp1 double mutant during meiosis, and found Klp2 deletion as the only suppressor. They convincingly show that klp2 deletion rescues cut7 pkl11 independent spindle formation in MI. The analysis of klp2 localization showed an increase in the total amount of Klp2 in MI compared to mitosis. Therefore they propose Klp2 as a new player in counteracting the Cut7- dependent outward pushing forces during MI.

Finally, they explore MT dynamics as another possible factor influencing the balance of forces produced by the spindle. They find that MTs are more dynamic during MI than during Mitosis. They test whether MT dynamics contribute to counteract Cut7 dependent outward forces during MI by altering MT dynamics with low doses of MBC. They found that whereas in wt cells low doses of MBC results in a slightly lower frequency of zygotes with 4 spores, in cut7Dpkl1D zygotes the same low doses of MBC, results in a higher frequency of zygotes producing 4 spores and thus suppresses cut7Dpkl1D phenotype.

In general I have enjoyed reading this manuscript. The data is clear and well presented and understanding the balances of forces necessary for proper spindle assembly and chromosome

© 2020. Published by The Company of Biologists under the terms of the Creative Commons Attribution License (https://creativecommons.org/licenses/by/4.0/).

segregation is an important problem in cell biology.

Reviewer 3 Comments for the Author:
The new version of the manuscript entitled "Kinesin-14s and microtubule dynamics define fission yeast mitotic and meiotic spindle assembly and elongation" by Loncar et al. has addressed all my comments. The manuscript has been improved significantly and I have no further concern.

We thank you for your positive review and recommendation.

In summary, we thank all three Reviewers for the positive recommendation. We have attempted to finalize the paper as per all suggestions. We believe it is now ready for acceptance and publication.

Cheers,
Ana Loncar & Phong Tran.

Third decision letter

MS ID#: JOCES/2019/240234

MS TITLE: Kinesin-14s and microtubule dynamics define distinct mitotic and meiotic spindle assembly and elongation.

AUTHORS: Ana Loncar, Sergio Rincon, Manuel Lera Ramirez, Anne Paoletti, and Phong Tran
ARTICLE TYPE: Research Article

I am happy to tell you that your manuscript has been accepted for publication in Journal of Cell Science, pending standard ethics checks.

© 2020. Published by The Company of Biologists under the terms of the Creative Commons Attribution License (https://creativecommons.org/licenses/by/4.0/).
